# Molecular Characterization of Mycoplasma pneumoniae Isolates in the United States from 2012 to 2018

**DOI:** 10.1128/JCM.00710-20

**Published:** 2020-09-22

**Authors:** L. Xiao, A. E. Ratliff, D. M. Crabb, E. Mixon, X. Qin, R. Selvarangan, Y.-W. Tang, X. Zheng, J. Dien Bard, T. Hong, M. Prichard, E. Brooks, S. Dallas, L. B. Duffy, K. B. Fowler, T. P. Atkinson, K. B. Waites

**Affiliations:** a Department of Medicine, University of Alabama at Birmingham, Birmingham, Alabama, USA; b Department of Pathology, University of Alabama at Birmingham, Birmingham, Alabama, USA; c Department of Pediatrics, University of Alabama at Birmingham, Birmingham, Alabama, USA; d Department of Laboratory Medicine and Pathology, Seattle Children’s Hospital, Seattle, Washington, USA; e Children’s Mercy Hospital, Kansas City, Missouri, USA; f Memorial Sloan Kettering Cancer Center, Weill Medical College of Cornell University, New York, New York, USA; g Ann & Robert H. Lurie Children’s Hospital of Chicago, Chicago, Illinois, USA; h Department of Pathology and Laboratory Medicine, Children’s Hospital Los Angeles, Keck School of Medicine, University of Southern California, Los Angeles, California, USA; i Department of Pathology, Hackensack University Medical Center, Hackensack, New Jersey, USA; j Departments of Pediatrics, University of Texas Health Science Center, San Antonio, Texas, USA; k Departments of Pathology, University of Texas Health Science Center, San Antonio, Texas, USA; Carter BloodCare & Baylor University Medical Center

**Keywords:** *Mycoplasma pneumoniae*, genotype, macrolide resistance, P1, variant, MLVA

## Abstract

Mycoplasma pneumoniae is a major cause of community-acquired pneumonia. There are limited data in the United States on the molecular epidemiological characteristics of M. pneumoniae. We collected 446 M. pneumoniae-positive specimens from 9 states between August 2012 and October 2018. Culture, antimicrobial susceptibility testing, P1 subtyping, and multilocus VNTR (variable-number tandem repeats) analysis (MLVA) were performed to characterize the isolates. Macrolide-resistant M. pneumoniae (MRMp) was detected in 37 (8.3%) specimens. P1 subtype 2 (P1-2) was the predominant P1 subtype (59.8%). P1 subtype distribution did not change significantly chronologically or geographically. The macrolide resistance rate in P1 subtype 1 (P1-1) samples was significantly higher than that in P1-2 (12.9% versus 5.5%). Six P1-2 variants were identified, including two novel types, and variant 2c was predominant (64.6%). P1-2 variants were distributed significantly differently among geographic regions. Classical P1-2 was more frequent in lower respiratory tract specimens and had longer *p1* trinucleotide repeats. Classical P1-2 was most common in MRMp (35.7%), while variant 2c was most common in macrolide-susceptible M. pneumoniae (67.5%). Fifteen MLVA types were identified; 3-5-6-2 (41.7%), 4-5-7-2 (35.3%), and 3-6-6-2 (16.6%) were the major types, and four MLVA clusters were delineated. The distribution of MLVA types varied significantly over time and geographic location. The predominant MLVA type switched from 4-5-7-2 to 3-5-6-2 in 2015. MLVA type was associated with P1 subtypes and P1-2 variant types but not with macrolide resistance. To investigate the M. pneumoniae genotype shift and its impact on clinical presentations, additional surveillance programs targeting more diverse populations and prolonged sampling times are required.

## Abstract (precis)

Mycoplasma pneumoniae is a major cause of community-acquired pneumonia. There are limited data in the United States on the molecular epidemiological characteristics of M. pneumoniae. We collected 446 M. pneumoniae-positive specimens from 9 states between August 2012 and October 2018. Culture, antimicrobial susceptibility testing, P1 subtyping, and multilocus VNTR (variable-number tandem repeats) analysis (MLVA) were performed to characterize the isolates.

## INTRODUCTION

Mycoplasma pneumoniae is a common bacterial pathogen in children, causing ∼8% of community-acquired pneumonia (CAP), and is responsible for ∼2% of CAP in adults during epidemic periods (1, 2). However, M. pneumoniae may cause up to 20 to 40% of cases of CAP in the general population and 70% in closed populations during epidemic periods that can occur every few years (3). M. pneumoniae can also cause extrapulmonary manifestations, including encephalitis, Stevens-Johnson syndrome, and hemolytic anemia.

Since 2010, M. pneumoniae outbreaks have been observed in Europe (3–5), the Middle East, and Asia (6–11). Macrolides are the first-line treatments for M. pneumoniae infections, but macrolide-resistant M. pneumoniae (MRMp) emerged in Japan in the early 2000s and is currently found in Asia, Europe, and North America (12–14). The resistance rate varies across regions, ranging from 1 to 30% in Europe to above 90% in parts of Asia (11). In the United States, MR prevalence ranged from 8.2% to 13.2% during 2006 to 2014 (15–17). A more recent surveillance across the United States found an overall MR prevalence of 7.5%, with rates at individual locations ranging from 1.9% to 21.7% (14).

M. pneumoniae genomes are extraordinarily similar and stable over time and geographic distance (18). Genome clustering indicates that there are two major clonal lineages (18, 19), corresponding to the two subtypes classified by variation in the *P1* adhesin gene (20). Based on sequence differences in the two repetitive elements (RepMP4 and RepMP2/3) in *p1*, M. pneumoniae can be divided into subtypes 1 (P1-1) and 2 (P1-2). Variants of each subtype can be generated from homologous recombination of RepMP4 and RepMP2/3 elements within *P1*, with other repetitive elements located across the genome (21). Other molecular typing methods, such as multilocus variable tandem repeat analysis (MLVA) (22) and multilocus sequence typing (MLST) (23), can also cluster M. pneumoniae isolates into different genotypes. However, the relationship between M. pneumoniae epidemics and the molecular subtypes remains unclear. Recent studies indicated that the incidence of MRMp is decreasing in some Asian countries, concurrent with a shift in the predominant genotypes (24–30). In 2017, the U.S. Centers for Disease Control and Prevention (CDC) sponsored a national surveillance program to determine the prevalence of MRMp infections in the United States between 2015 and 2018 (14). We performed molecular genotyping and clinical character analysis on patients and samples collected in this study and extended the genotyping back to 2012 by retrieving samples from a previous study targeting the same geographic regions (17) and from existing laboratory collections at the UAB Diagnostic Mycoplasma Laboratory.

## MATERIALS AND METHODS

### Clinical specimens and M. pneumoniae isolates.

A total of 446 respiratory specimens were collected between August 2012 and October 2018. Among them, 360 were from the CDC surveillance program collected from 2015 to 2018 (14), 71 were from a previous study collected from August 2012 to April 2014 (17), and 15 were from routine laboratory accessions during the 7-year period. Each specimen was from a unique patient. The geographic regions represented included nine U.S. states (AL, CA, CO, IL, MO, NJ, NY, TX, and WA). Specimens included those from the upper respiratory tract (URT; including nasopharyngeal or oropharyngeal swabs/aspirates, nasal aspirate/washes, midturbinate swabs, and throat swabs), lower respiratory tract (LRT; including bronchoalveolar lavage [BAL] fluid, tracheal aspirate, and sputum), and blood (see Table S1 in the supplemental material).

All specimens were tested by real-time PCR (31) using a Roche LightCycler 480 (Roche Diagnostics, Indianapolis, IN) to verify the M. pneumoniae positivity and cultured using the SP4 broth-to-agar method (32). Specimens were also tested by real-time PCR (33) to detect point mutations in the *23S rRNA* gene associated with MR (11, 33). Amplicons of the mutants were sequenced to determine the exact mutations (14).

### Antimicrobial susceptibility testing.

The minimum inhibitory concentration (MIC) for erythromycin was determined by standardized methods established by the Clinical and Laboratory Standards Institute (CLSI). Erythromycin MICs of ≥1 μg/ml were considered resistant (34).

### P1 subtyping.

DNA from M. pneumoniae isolates and original specimens was purified using MagNA Pure 2.0 (Roche Diagnostics, Indianapolis, IL). Portions of the *P1* gene spanning the repetitive sequences RepMP4 and RepMP2/3 were amplified using primer pairs ADH1/ADH2 (35) and Mp5f/M16r (36), respectively. Conventional PCR was performed on the Veriti 96-well thermal cycler (Applied Biosystems, Foster City, CA) with a 25-μl PCR volume containing 0.4 μmol/liter of each primer, 2.5 μl of 10× AccuPrime Pfx reaction mix (Thermo Fisher, Fremont, CA), 0.5 U of AccuPrime Pfx DNA polymerase, and 2 μl of template DNA. Amplification conditions were 95°C for 2 min; 5 cycles of 95°C for 15 s, 60°C for 15 s, and 68°C for 2.5 min; and 40 cycles of 95°C for 15 s, 55°C for 15 s, and 68°C for 2.5 min. For samples that failed the first amplification, nested PCRs were performed to assist the discrimination of P1 subtypes and variants. Nested primer pair Mp705/ADH3R was used for the ADH1/ADH2 amplicon; Mp11f/Mp14r was for the Mp5f/Mp16r amplicon (36) (Table S2). Amplicons were sequenced by Sanger sequencing at the UAB Heflin Genomics Center and analyzed using CLC Genomics Workbench 20 (Qiagen, Redwood City, CA).

### MLVA.

The 4-locus MLVA typing scheme (Mpn13 to Mpn16) was used for this study (37). Conventional PCR was carried out using primer pairs as published previously (22). Nested PCR was used for samples that failed the first amplification (38). Amplicons were sequenced and analyzed as described above.

### Data analyses.

Reference sequences of P1 subtypes and variants were downloaded from NCBI: P1 subtype 1 (P1-1, U00089.2:180858.185741, strain M129), P1 subtype 2 (P1-2, CP002077.1:179293-184197, strain FH), variant 1 (AF290000.1), variant 2a (AP012303.1:179359-184257), variant 2b (AP017318.1:179335-184254), variant 2bv (MK330954.1), variant 2c (AP017319.1:179294-184195), variant 2c2 (JN048894.1), variant 2d (EF656612.1), variant 2f (LC311244.1), and variant 2g (LC385984.1). There is conflict in the description of variant 2e in previous reports (25, 39–41). Sequence MK330954.1 was designated variant 2e (40) and is identical to the sequence reported as 2bv (41). Since this sequence is very similar to variant 2b (with a 12-bp deletion), in this study, the MK330954.1 sequence is classified as 2bv. Variant 2e was renamed from V2d (strain Mp100) (25, 39). Since its sequence was not deposited in public databases, the core corresponding sequence was extracted from the original reference and used as reference 2e (39). Assembled *P1* and MLVA sequences were aligned to the reference sequences for comparison, and P1 subtypes, P1-2 variant types, and MLVA types were assigned to each specimen. The copy number of the trinucleotide (AGT) VNTR within the *P1* gene was also counted. MLVA typing data were uploaded into the BioNumerics software 7.6 (Applied Maths, Austin, TX). A dendrogram was generated based on the categorical coefficient and the algorithm of unweighted pair group method with arithmetic mean (UPGMA). A cutoff value of 67% similarity was applied to define MLVA clusters. Minimum spanning trees (MSTs) were constructed using the categorical coefficient and the priority rule for standard MST with single- and double-locus variants. The discriminatory power was calculated using the Hunter-Gaston diversity index (HGDI) (42).

### Statistical analyses.

Chi-square or Fisher’s exact test was used to analyze the correlation of genotypes and their relationships with factors such as time, geographic region, specimen types, and MR conditions. Student’s *t* test or one-way analysis of variance (ANOVA) coupled with Tukey's honestly significant difference (HSD) *post hoc* test was used to compare the mean *p1* VNTR copy number in different groups. A *P* value of <0.05 was considered statistically significant, except for the cases of Bonferroni correction. SAS 9.4 (SAS Institute Inc., Cary, NC) and SPSS 26 (IBM Corp., Armonk, NY) were used for statistical analysis.

### Human subject considerations.

Institutional Review Board approvals were obtained at UAB and all clinical test sites. Any identifying information was removed from specimens before shipment to UAB. Clinical data were entered electronically into a computerized database (REDCap) (43) and linked to laboratory data only by study numbers.

### Data availability.

The partial sequences of the P1 gene of strain 72255 (P1-2 variant 2h) and strain 73192 (P1-2 variant 2i) were deposited in GenBank. The accession numbers are MT319404 (for strain 72255) and MT319405 (for strain 73192).

## RESULTS

### Specimens, M. pneumoniae culture, and antimicrobial susceptibility testing.

**FIG 1:**
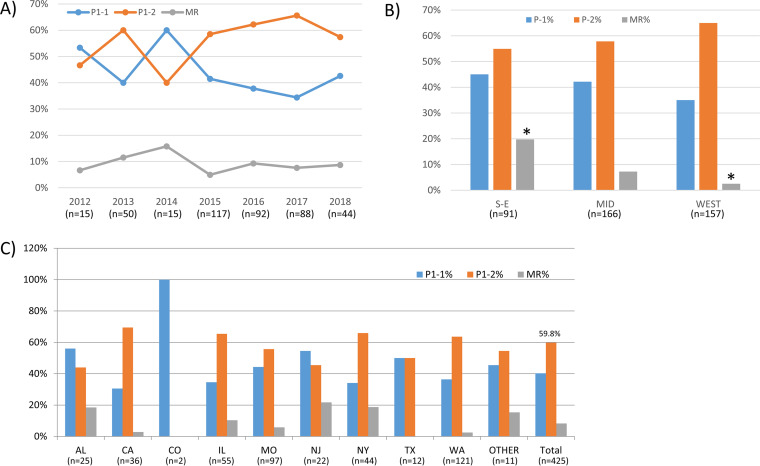
P1 subtype and MRMp distribution. (A) Distribution of P1 subtypes (*P* = 0.587) and MRMp (*P* = 0.651) over time. (B) Distribution of P1 subtypes (*P* = 0.234) and MRMp (*P* < 0.001) in the South/East (S-E), Midwest (MID), and West regions of the United States. (C) Distribution of P1 subtypes (*P* = 0.197) and MRMp (*P* = 0.008) in different states. An asterisk indicates significant difference after Bonferroni correction.

Among 446 specimens, 420 (94.2%) were URT and 24 (5.4%) were LRT (see Table S1 in the supplemental material). The specimen proportions from different anatomic sites varied significantly among regions and years (Table S3). More LRT specimens were collected in the South/East (17/97, 17.5%) and in year 2014 (4/18, 22.2%) than in other regions or years. There were 323 specimens successfully cultured, and MICs for erythromycin were available for 317 isolates (Table 1). MICs ranged from 0.001 to >256 μg/ml, with the majority (294 isolates, 92.7%) being less than 0.008 μg/ml. There were 23 (7.3%) isolates having a MIC of >8 μg/ml, corresponding to the PCR results detecting *23S rRNA* gene mutations. A2063G (M. pneumoniae numbering, 32/37, 86.5%) was the most common mutation detected in the *23S rRNA* gene. The prevalence of MRMp by PCR in the United States between 2012 and 2018 was 8.3% (37/446) and did not vary with time (*P* = 0.651) (Table 1 and Fig. 1A). The MR rate decreased from 19.4% (19/98) in the South/East (AL, NJ, and NY) to 2.5% (4/158) in the West (CA and WA) (*P* < 0.001) (Fig. 1B). MR rate was also different among the states (*P* = 0.002) (Fig. 1C). NJ was highest (5/23, 21.7%), while no resistance was observed in TX and CO. The MR rate in LRT specimens (3/24, 12.5%) was higher than that in URT specimens (34/420, 8.1%), without a significant difference (*P* = 0.439) (Table S3).

**TABLE 1:** Summary of P1 typing results and macrolide-susceptibility status of M. pneumoniae specimens^*a*^

Summary parameter	Total no. of specimens	No. of specimens for:															
		P1 subtype			P1-2 variant									MS		MR	
		P1-1	P1-2	NT	Classical 2	2a	2a/2c	2b	2c	2bv	2h	2i	No. Variants	PCR	Culture	PCR	Culture
Geographic regions																	
AL	27	14	11	2	6	1	0	1	2	1	0	0	5	22	13	5	4
CA	36	11	25	0	1	0	2	2	18	2	0	0	24	35	23	1	1
CO	2	2	0	0	0	0	0	0	0	0	0	0	0	2	2	0	0
IL	58	19	36	3	4	1	1	0	26	4	0	0	32	52	40	6	3
MO	104	43	54	7	8	4	0	7	33	2	0	0	46	98	51	6	5
NJ	23	12	10	1	0	0	0	0	10	0	0	0	10	18	13	5	3
NY	48	15	29	4	9	0	0	1	16	1	2	0	20	39	28	9	4
TX	13	6	6	1	1	0	0	0	3	1	0	1	5	13	11	0	0
WA	122	44	77	1	9	1	0	7	52	8	0	0	68	119	106	3	2
Other	13	5	6	2	0	1	0	0	4	1	0	0	6	11	7	2	1
Total (%)	446	171 (40.2)	254 (59.8)	21 (4.7)	38 (15.0)	8 (3.1)	3 (1.2)	18 (7.1)	164 (64.6)	20 (7.9)	2 (0.8)	1 (0.4)	216 (85.0)	409 (91.7)	294 (92.7)	37 (8.3)	23 (7.3)
Year																	
2012	15	8	7	0	1	0	0	0	6	0	0	0	6	14	13	1	1
2013	52	20	30	2	6	1	0	4	17	2	0	0	24	46	37	6	5
2014	19	9	6	4	0	1	0	1	3	1	0	0	6	16	10	3	2
2015	121	48	69	4	9	2	1	10	41	6	0	0	60	115	77	6	2
2016	97	35	57	5	7	4	0	0	40	6	0	0	50	88	58	9	4
2017	92	30	58	4	11	0	2	3	38	2	2	0	47	85	63	7	6
2018	46	19	25	2	2	0	0	0	19	3	0	1	23	42	34	4	3
Year not specified	4	2	2	0	2	0	0	0	0	0	0	0	0	3	2	1	0
Total	446	171 (40.2)	254 (59.8)	21 (4.7)	38 (15.0)	8 (3.1)	3 (1.2)	18 (7.1)	164 (64.6)	20 (7.9)	2 (0.8)	1 (0.4)	216 (85.0)	409 (91.7)	294 (92.7)	37 (8.3)	23 (7.3)

a NT, not typed; MS, macrolide susceptible; MR, macrolide resistant.

### M. pneumoniae P1 subtyping.

**FIG 2:**
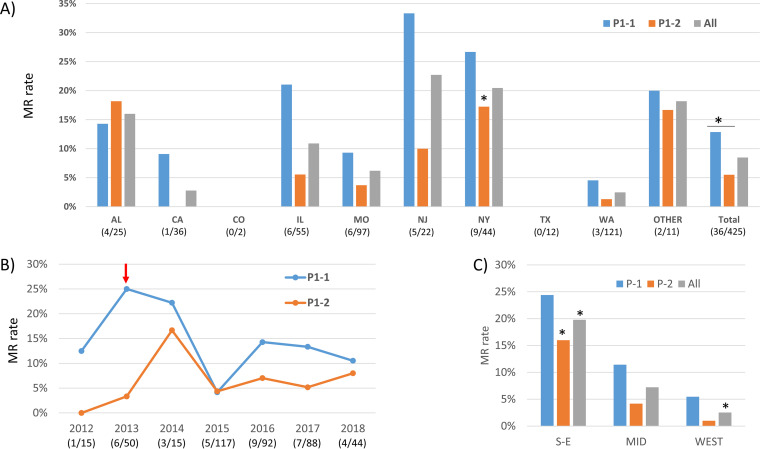
Macrolide resistance in P1 subtypes (*P* = 0.012 overall). (A) Macrolide resistance in P1-1 (*P* = 0.165) and P1-2 (*P* = 0.024) subtypes in different states. (B) Macrolide resistance rate in P1-1 (*P* = 0.330) and P1-2 (*P* = 0.830) subtypes over time. (C) Macrolide-resistant rate in P1-1 (*P* = 0.02) and P1-2 (*P* < 0.001) in the South/East (S-E), Midwest (MID), and West regions of the United States. An asterisk indicates significant difference (*P* < 0.05 after Bonferroni correction).

A total of 425 specimens were successfully analyzed for P1 subtypes. P1-2 was the overall dominant subtype (254/425, 59.8%) (Table 1). Overall distribution of P1 subtypes did not change significantly over the 7-year study period (*P* = 0.587) (Fig. 1A). However, P1-1 was more common in 2012 and 2014. After 2015, P1-2 became consistently dominant. In 2018, the proportion of P1-2 started to decrease. The distribution did not vary significantly across the states (*P* = 0.197) (Fig. 1C) or the three general regions (*P* = 0.234) (Fig. 1B). The P1-2 proportion was highest in CA (25/36, 69.4%), while P1-1 was slightly more common in AL (14/25, 56.0%) and NJ (12/22, 54.5%). MR was more common in P1-1 (22/149, 12.9%) than in P1-2 (14/240, 5.5%; *P* = 0.012) (Fig. 2A). There was no significant difference in MR rates in P1 subtypes over time, except in 2013 the MR rate was significantly higher in P1-1 (5/20, 25%) than in P1-2 (1/30, 3.3%) (*P* = 0.032) (Fig. 2B). Within P1-1, the MR rate varied from 0% in TX and CO to 33.3% (4/12) in NJ, 24.4% (10/41) in the South/East, and 5.5% (3/55) in the West (Fig. 2A). The difference was significant only when considering the three general regions (*P* = 0.02) (Fig. 2C). In P1-2, the MR rate varied from 0% in CA and TX to 18.2% in AL (2/11) (Fig. 2A). The difference was significant across the states (*P* = 0.024) (Fig. 2A) and in the three general regions (*P* < 0.001) (Fig. 2C). In AL, the MR rate was higher in P1-2 than that in P1-1 (2/12, 14.3%), which differed from other states (Fig. 2A). Although not significantly different, P1-1 (13/22, 59.1%) was more prevalent in LRT specimens, while P1-2 was more common in URT specimens (245/401, 61.1%) (*P* = 0.074) (Table S3).

### P1 subtype variants.

**FIG 3:**
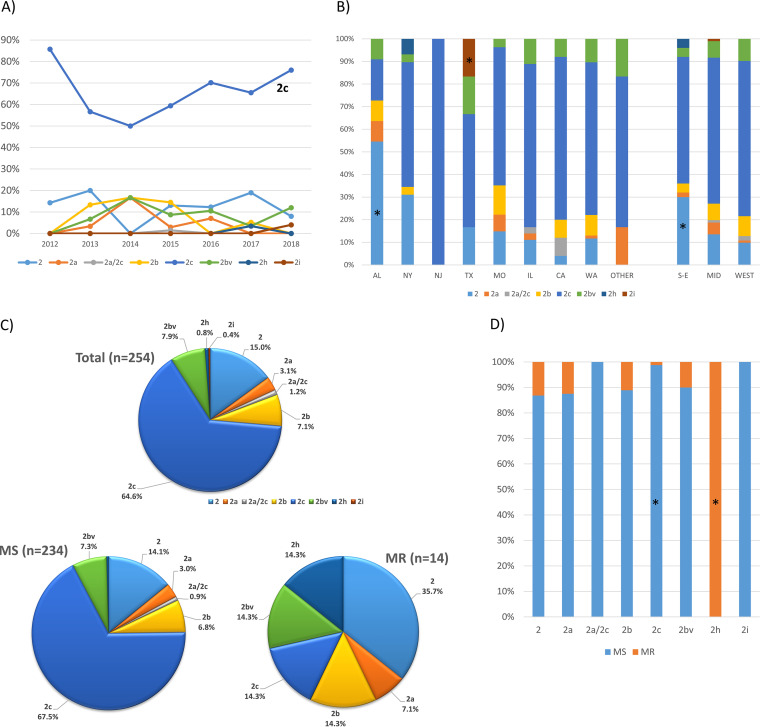
P1-2 variant distribution. (A) P1-2 variant distribution over time (*P* = 0.058). (B) P1-2 variant distribution in states (*P* < 0.001) and in the South/East, Midwest, and West regions (*P* = 0.005) of the United States. (C) P1-2 variant distribution in overall P1-2 specimens, macrolide-susceptible and macrolide-resistant (*P* < 0.001) specimens. (D) Macrolide-resistance rate in P1-2 variants (*P* < 0.001). An asterisk indicates significant difference after Bonferroni correction.

No P1-1 variants were detected. Six P1-2 variants, including two novel types, 2h and 2i, were identified in the 254 successfully analyzed P1-2 samples (Table 1 and Fig. 3). There were 3 specimens identified as variant 2a or 2c (2a/2c) due to failed amplification of the RepMP4 region. Overall, variant 2c was the major P1-2 variant, comprising 64.6% (164/254) of all P1-2 samples, followed by classical type 2 (38/254, 15.0%) and variant 2bv (20/254, 7.9%) (Table 1 and Fig. 3C). The distribution of the variants changed temporally, with the difference approaching significance (*P* = 0.058) (Fig. 3A). The proportion of variant 2c decreased from 85.7% to 50.0% from 2012 to 2014 and then slowly increased to 76.0% by 2018. The total proportion of all other variants was below 20% during the study. The novel variants were from two states: two variants of 2h were from NY, and one variant, 2i, was from TX. Variant distribution was different across all states (*P* < 0.001) (Fig. 3B). The major variant in AL was classical P1-2 (6/11, 54.5%) instead of 2c. Most of the states had 4 to 5 variant types, while NJ only had one type, 2c. The variant distribution was also different from the South/East to the West (*P* = 0.005) (Fig. 3B), and classical P1-2 was significantly more common in the South/East (15/50, 30.0%). Between macrolide-susceptible (MS) M. pneumoniae and MRMp groups, the variant distribution was significantly different (*P* < 0.001) (Fig. 3C). Variant 2c was most common in MSMps (158/234, 67.5%), while classical P1-2 was most common in MRMps (5/14, 35.7%). Both 2h variants (2/2, 100%) were MR. MR prevalence in different variants ranged from 0% in 2i to 100% in variant 2h (2/2) (Fig. 3D), with the majority ranging from 10% to 13.2% (2bv, 2b, 2a, and classical P1-2). MR in variant 2c was very low (2/164, 1.2%). P1-2 variants were distributed significantly differently in URT and LRT specimens (Table S3) (*P* = 0.026). Classical P1-2 was significantly more common in LRT specimens (5/9, 55.6%).

Both variants 2h and 2i had the element g (RepMP4-g) sequence in the RepMP4 region, which was the same as variants 2c and 2f (21, 25) (Fig. S1; GenBank accession numbers MT319404 [2h] and MT319405 [2i]). In the RepMP2/3 region, variant 2h had a 70-bp fragment (corresponding to nucleotides 2742 to 2799 in FH *P1* gene), probably from homologous recombination with element b (RepMP2/3-b), although there are minor mismatches between these sequences, while variant 2i contained a 187-bp fragment (corresponding to nucleotide 2730 to 2901 in FH *P1* gene) from homologous recombination with element j (RepMP2/3-j).

### *p1* VNTR.

Among the 274 specimens available for *P1* VNTR analysis, the copy number ranged from 5 to 21, with a mean of 7.8 ± 1.9 and a median of 7.0 (interquartile range, 2.0) (Table 2). There was no significant difference in the mean copy numbers among regions, years, specimen types, MR conditions, and P1 subtypes, except P1-2 variants (*P* = 0.01). Classical P1-2 had longer VNTRs (mean, 8.6 ± 1.0; median, 8.0) than variants 2a and 2c (mean, 7.5 ± 1.6; median, 7.0). Considering the distribution, significant differences were noticed among specimen types, P1 subtypes, and P1-2 variant groups (Table S4 and Fig. S2). The specimen with the longest VNTR (21 copies) was from LRT. The most common VNTR copy number in P1-1 was 9 (22/101, 21.8%); in P1-2, the number was 7 (56/173, 32.4%). The shortest VNTR (5 copies) was significantly more prevalent in P1-1 (9/101, 8.9%) than P1-2 (1/173, 0.6%). The HGDI of *p1* VNTR on the typed specimens was 0.8194.

**TABLE 2:** Comparison of the means of the P1 VNTR copy numbers^*a*^

Category^*b*^	No. of specimens	Copy no.			*P* value
		Mean ± SD	Median	Range	
All specimens	274	7.9 ± 1.9	7.0	5–21	
Region					0.17
South/East	66	8.2 ± 2.6	7.0	5–21	
Middle	85	7.8 ± 1.7	8.0	5–14	
West	119	7.7 ± 1.6	7.0	5–14	
Total	270	7.9 ± 1.9	7.0	5–21	
Year					0.665
2012	3	8.0 ± 1.7	7.0	7–10	
2013	15	7.6 ± 2.0	7.0	6–14	
2014	5	7.6 ± 2.1	7.0	6–11	
2015	80	7.8 ± 1.3	8.0	5–11	
2016	60	7.9 ± 1.5	8.0	5–13	
2017	71	7.5 ± 1.8	7.0	5–14	
2018	38	8.2 ± 2.6	7.0	5–14	
Total	272	7.8 ± 1.8	7.0	5–14	
Specimen type					0.478
URT	263	7.8 ± 1.8	7.0	5–14	
LRT	11	8.8 ± 4.5	8.0	5–21	
Total	274	7.8 ± 1.9	7.0	5–21	
MR mutation					0.897
MR	19	7.9 ± 1.8	8.0	5–12	
WT	255	7.9 ± 2.0	7.0	5–21	
Total	274	7.9 ± 1.9	7.0	5–21	
P1 subtype					0.21
P1-1	101	8.1 ± 2.2	8.0	5–21	
P1-2	173	7.7 ± 1.7	7.0	5–14	
Total	274	7.9 ± 1.9	7.0	5–21	
P1-2 variant					0.01
Classical 2^*^	27	8.6 ± 1.0	8.0	6–13	
2a and 2c^*^	123	7.5 ± 1.6	7.0	5–14	
2b and 2bv	20	8.2 ± 1.8	8.0	6–12	
2h and 2i	3	7.0 ± 1.7	6.0	6–9	
Total	173	7.7 ± 1.7	7.0	5–14	
MLVA type					0.012
3-5-6-2^#^	126	7.5 ± 1.6	7.0	5–14	
3-6-6-2^#^	46	8.5 ± 1.9	8.0	6–13	
4-5-7-2	88	8.1 ± 2.2	8.0	5–21	
Others	14	7.9 ± 2.5	7.5	5–14	
Total	274	7.9 ± 1.9	7.0	5–21	

a URT, upper respiratory tract; LRT, lower respiratory tract; MR, macrolide resistance; WT, wild type.

b “*” and “#” indicate significant difference between the groups.

### MLVA typing.

**FIG 4:**
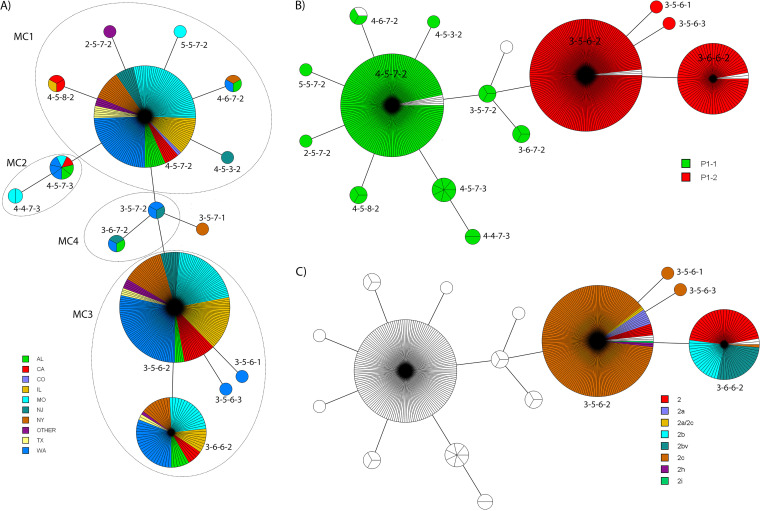
Minimum spanning tree (MST) of the 428 M. pneumoniae strains based on MLVA types. The MLVA data were analyzed by BioNumerics. Clustering of MLVA profiles was done using a categorical coefficient. Each circle represents one MLVA type and the size of the circle is proportional to the number of isolates. Each line represents one allelic change and the distance between MLVA types corresponds to the total length of lines. (A) MST with colors based on the states. Circles with dashed lines delineate MLVA clusters (MCs). (B) MST with colors based on P1 subtypes. (C) MST with colors based on P1-2 variants.

There were 428 specimens successfully analyzed for MLVA types, and 15 MLVA types were identified (Table 3). The HGDI of the MLVA typing scheme in these specimens is 0.6742. The major MLVA types were 3-5-6-2 (179/428, 41.8%), 4-5-7-2 (151/428, 35.3%), and 3-6-6-2 (71/728, 16.6%). The 428 specimens were clustered into 2 major lineages or 4 MLVA clusters (MCs) and one singleton based on a cutoff value of 67% genetic similarity (Fig. 4A and Fig. S3). The biggest cluster was MC3, consisting of 252 (58.9%) specimens and 4 MLVA types featuring 3-X-6-X. MC1 had 160 (37.4%) specimens and 6 MLVA types, with 4-5-7-2 most frequently identified. MC4 was the transitioning cluster located between MC1 and MC3, consisting of 2 MLVA types (3-5-7-2 and 3-6-7-2). MC2 was branched from MC1, containing MLVA types 4-5-7-3 and 4-4-7-3. The singleton MLVA type 3-5-7-1 was branched from MC4.

**TABLE 3:** Summary of MLVA types

Summary parameter	No. of specimens	No. of MLVA type:															
		2-5-7-2	3-5-6-1	3-5-6-2	3-5-6-3	3-5-7-1	3-5-7-2	3-6-6-2	3-6-7-2	4-4-7-3	4-5-3-2	4-5-7-2	4-5-7-3	4-5-8-2	4-6-7-2	5-5-7-2	NT^*a*^
Geographic region																	
AL	27	0	0	5	0	0	0	6	1	0	0	10	2	0	1	0	2
CA	36	0	0	17	0	0	0	5	0	0	0	7	1	2	0	0	4
CO	2	0	0	0	0	0	0	0	0	0	0	2	0	0	0	0	0
IL	58	0	0	28	0	0	0	8	0	0	0	18	0	1	0	0	3
MO	104	0	0	37	0	0	0	17	0	2	0	44	1	0	0	1	2
NJ	23	0	0	10	0	0	1	0	1	0	1	9	0	0	0	0	1
NY	48	0	0	22	0	1	0	10	0	0	0	14	0	0	1	0	0
TX	13	0	0	4	0	0	0	2	0	0	0	6	0	0	0	0	1
WA	122	0	1	51	1	0	2	22	1	0	0	37	3	0	1	0	3
Other	13	1	0	5	0	0	0	1	0	0	0	4	0	0	0	0	2
Total (%)	446	1 (0.2)	1 (0.2)	179 (41.8)	1 (0.2)	1 (0.2)	3 (0.7)	71 (16.6)	3 (0.7)	2 (0.5)	1 (0.2)	151 (35.3)	7 (1.6)	3 (0.7)	3 (0.7)	1 (0.2)	18 (4.0)
Year																	
2012	15	0	1	5	0	0	0	1	0	0	0	6	1	0	0	1	0
2013	52	0	0	16	0	0	1	12	0	0	0	19	0	0	0	0	4
2014	19	1	0	5	0	0	0	2	0	0	0	8	0	0	0	0	3
2015	121	0	0	45	0	0	0	22	0	0	0	45	3	1	1	0	4
2016	97	0	0	44	0	1	0	13	1	2	0	30	2	0	1	0	3
2017	92	0	0	43	1	0	1	15	1	0	0	26	1	1	1	0	2
2018	46	0	0	20	0	0	1	5	1	0	1	15	0	1	0	0	2
Year not specified	4	0	0	1	0	0	0	1	0	0	0	2	0	0	0	0	0
Total (%)	446	1 (0.2)	1 (0.2)	179 (41.8)	1 (0.2)	1 (0.2)	3 (0.7)	71 (16.6)	3 (0.7)	2 (0.5)	1 (0.2)	151 (35.3)	7 (1.6)	3 (0.7)	3 (0.7)	1 (0.2)	18 (4.0)

a NT, not typed.

**FIG 5:**
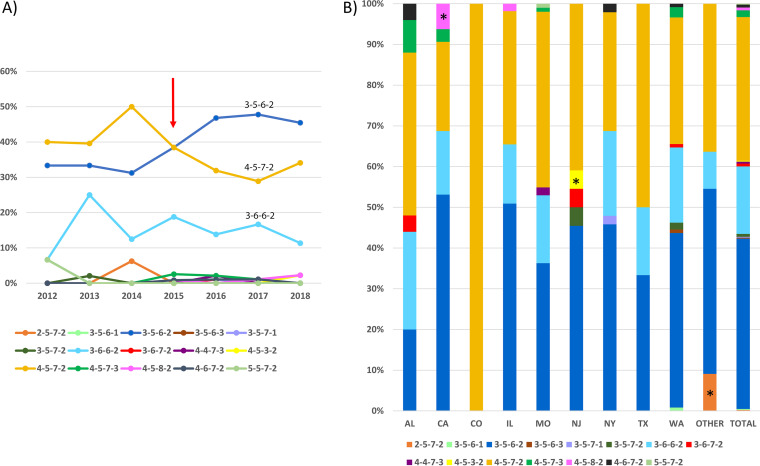
MLVA types. (A) MLVA type distribution over years (*P* = 0.01). (B) MLVA type distribution in different states (*P* = 0.05). An asterisk indicates significant difference after Bonferroni correction.

The distribution of MLVA types changed significantly over time (*P* = 0.01) (Fig. 5A). MLVA type 4-5-7-2 was predominant before 2015. After 2015, the predominant MLVA type switched to 3-5-6-2. The percentage of MLVA type 3-6-6-2 was constant over the surveillance period. The aggregate of other MLVA types was less than 10%, except in 2012, in which the combined total was approximately 20%. The distribution of MLVA types also differed geographically (*P* = 0.05) (Fig. 4A and 5B). While MLVA type 3-5-6-2 was predominant overall, type 4-5-7-2 was more common in four states (MO, TX, AL, and CO). The number of MLVA types in each state was different. WA had the most types (nine), while CO had only one. There was no difference in the distribution of MLVA types among the three major regions (*P* = 0.211) or between URT and LRT specimens (*P* = 0.792) (Table S3).

MLVA types were significantly associated with P1 subtypes (*P* < 0.001) (Table 4, Fig. 4B, and Fig. S3). The two major MLVA cluster lineages represented P1-1 and P1-2 subtypes (Fig. S3). MLVA types 4-5-7-2 (146/169, 86.4%) and 4-5-7-3 (7/169, 4.1%) constituted over 90% of all MLVA types in P1-1, and all of the 169 P1-1 specimens are in MLVA clusters MC1, MC2, and MC4 (Fig. 4B). On the other hand, MLVA types 3-5-6-2 (176/247, 71.3%) and 3-6-6-2 (69/247, 27.9%) predominated in P1-2, and all 247 P1-2 specimens are in MLVA cluster MC3 (Fig. 4B). More MLVA types were found in P1-1 than in P1-2 (10 versus 4). Within P1-2, the variant types were also significantly associated with MLVA types (*P* < 0.001) (Table 5 and Fig. 4C). Variant 2c was predominant in MLVA type 3-5-6-2 (157/176, 89.2%). The classical type 2 (32/69, 46.4%), 2b (17/69, 24.6%), and 2bv (19/69, 27.5%) were the major variants in MLVA type 3-6-6-2. The mean copy number of *p1* VNTR was different among the MLVA groups (*P* = 0.012) (Table 2). MLVA type 3-5-6-2 (mean, 7.5 ± 1.6; median, 7.0) had fewer copies than 3-6-6-2 (mean, 8.5 ± 1.9; median, 8.0). The distribution of the *p1* VNTR was also significantly different among the MLVA types (*P* < 0.001) (Table S4 and Fig. S2C).

**TABLE 4:** Distribution of MLVA types within P1 subtypes

P1 subtype and MLVA type^*a*^	No. of specimens	Percentage
P1-1 (*n* = 169)		
2-5-7-2	1	0.6
3-5-7-2	3	1.8
3-6-7-2	3	1.8
4-4-7-3	2	1.2
4-5-3-2	1	0.6
4-5-7-2*	146	86.4
4-5-7-3*	7	4.1
4-5-8-2	3	1.8
4-6-7-2	2	1.2
5-5-7-2	1	0.6
P1-2 (*n* = 247)		
3-5-6-1	1	0.4
3-5-6-2*	176	71.3
3-5-6-3	1	0.4
3-6-6-2*	69	27.9

a Values marked by asterisks are significant after Bonferroni correction.

**TABLE 5:** Distribution of P1-2 variants within MLVA types

MLVA type and P1-2 variant^*a*^	No. of specimens	Percentage
3-5-6-1 (*n* = 1)		
2c	1	100.0
3-5-6-2 (*n* = 176)		
2	6	3.4
2a	8	4.5
2a/2c	2	1.1
2c*	157	89.2
2h	2	1.1
2i	1	0.6
3-5-6-3 (*n* = 1)		
2c	1	100.0
3-6-6-2 (*n* = 69)		
2*	32	46.4
2b*	17	24.6
2c	1	1.4
2bv*	19	27.5

a Values marked by asterisks are significant after Bonferroni correction.

**FIG 6:**
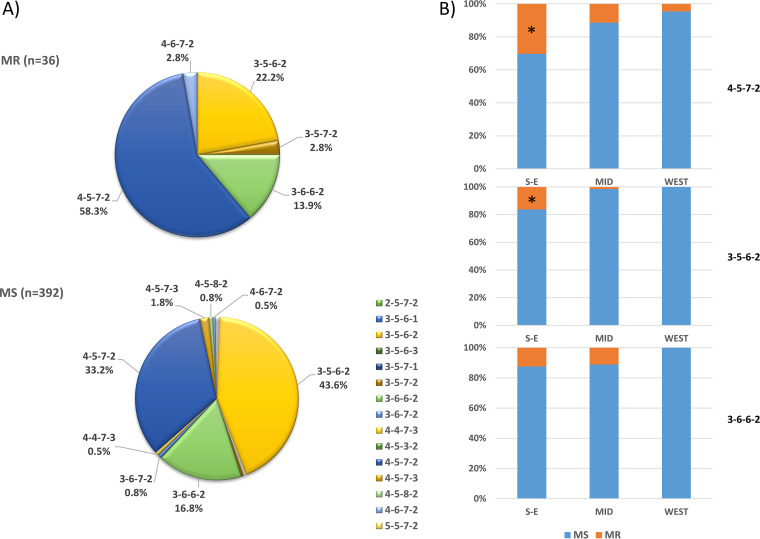
Macrolide resistance in MLVA types. (A) The overall distribution of MLVA types in MR and MS specimens (*P* = 0.286). (B) MR and MS specimens in MVLA types 4-5-7-2 (*P* = 0.004), 3-5-6-2 (*P* < 0.001), and 3-6-6-2 (*P* = 0.182) in the South/East, Midwest, and West regions of the United States. An asterisk indicates significant difference after Bonferroni correction.

There were 5 MLVA types (3-5-6-2, 3-6-6-2, 3-5-7-2, 4-5-7-2, and 4-6-7-2) in MRMp, while all 15 types were present in MSMp (Fig. 6A). MLVA type 4-5-7-2 was most common in MRMp (21/36, 58.3%), and 3-5-6-2 was most prevalent in MSMp (171/392, 43.6%). In other words, MR prevalence was higher in MLVA type 4-5-7-2 (21/151, 13.9%) than in 3-6-6-2 (5/71, 7.0%) and 3-5-6-2 (8/179, 4.5%). However, there was no significant association between MR and MLVA type (*P* = 0.286). MR prevalence in MLVA type 4-5-7-2 and 3-5-6-2 was different among the states (*P* = 0.044 and 0.001, respectively) (Fig. S4). MR 4-5-7-2 was found in all states except TX and CO and was more common in NJ and NY (4/9, 44.4% and 4/14, 28.6%). MR 3-5-6-2 was found in only 3 states, NY (5/22, 22.7%), NJ (1/10, 10.0%), and IL (1/28, 3.6%). The prevalence of MR in MLVA types 4-5-7-2 and 3-5-6-2 was also different from the South/East to the West (*P* values of 0.004 and <0.001, respectively) (Fig. 6B). The MR rate in MLVA type 4-5-7-2 was 30.3% (10/33), 11.4% (8/70), and 4.5% (2/44) in the South/East, Midwest, and West, respectively; for MLVA type 3-5-6-2, the prevalence in the same regions was 16.4% (6/37), 1.4% (1/69), and 0.0% (0/68), respectively.

## DISCUSSION

This study analyzed the molecular characteristics of 446 M. pneumoniae specimens collected in the United States from 2012 to 2018. Results showed that P1-2 was the major P1 subtype (59.8%), and six P1-2 variants were identified, with variant 2c being predominant (64.6%). Among 15 MLVA types, 3-5-6-2 (41.7%) was the most common type. The predominant MLVA type switched from 4-5-7-2 to 3-5-6-2 in 2015. Distribution of P1-2 variants and MLVA types varied geographically. MR was significantly more common in P1-1. MLVA type was associated with P1 subtypes and P1-2 variant types but not with MR.

Both P1 subtypes occurred in the United States during 2012 to 2018, and P1-2 was slightly predominant (59.8%). This result contrasts somewhat with the previous observation that P1-1 was the major M. pneumoniae subtype (57%) in the United States from 2006 to 2013 (16). However, this report also noted that the proportion of P1-1 was starting to decrease between 2011 and 2013 (16). This difference probably reflects the natural shifting of P1 subtypes during the two investigational periods. Kenri et al. reported that a type shift phenomenon occurs every 8 to 10 years, and a shift from one type to another required a transition period of 2 to 3 years in Japan (44). Similarly, in this study, there was a transitional stage between 2012 and 2014 before P1-2 stabilized as the predominant subtype (Fig. 1A). Other reports have indicated that there has been a recent subtype shift occurring worldwide beginning around 2013: a shift from P1-1 to P1-2 in China (45, 46), Japan (25, 47, 48), and South Korea (28) and a shift from P1-2 to P1-1 in Slovenia (49). Although P1-2 was predominant overall in this study, it never achieved absolute predominance (i.e., >90%) as in the previous shifts in Asia (44, 46). Both subtypes circulated in the communities in a comparable ratio across the country for several years. Similarly, in France, after a subtype shift from P1-1 to P1-2 in 1996 to 1997, both subtypes were present in about the same proportion from 1998 to 2006 (50). In Germany, both subtypes were circulating without a clear trend of dominance between 2003 and 2012 (3). Cocirculation of both subtypes with a comparable ratio may be due to herd immunity. The proportion of immunity to the dominant and nondominant strains may be similar in situations where there is no clear predominance of either. Recently, a mathematical model for two strains of comparable activity levels that cocirculate within a network-structured population has been proposed that may explain this phenomenon (51). In this model, strain dominance alternates and oscillates during the epidemic cycle without a complete replacement of one particular strain. Interestingly, Zhao et al. reported from Beijing that after the subtype shift from P1-1 to P1-2 in 2013, the ratio of the two subtypes did not increase as quickly as expected but slowly reached near equivalence during 2014 to 2016 (27). A similar trend was also observed in Japan (25). In this study, after dominating for 4 years, the proportion of P1-2 appeared to decrease in 2018. We predict that the dominant P1 subtype in the United States will shift from P1-2 to P1-1 beginning in 2018 and that the continuing shift will last for several years without establishing an absolute dominance of P1-1.

This study identified six P1-2 variants. P1 subtype variants (1a, 2a-c, 2c2, and 2d to g) have been reported from different regions since 1999 (25, 36, 39, 40, 50, 52–55). Studies have revealed that P1-2 variants have been evolving for years (25, 28, 40, 44–46). P1-2 variants were rare before early 2000s (40, 44, 56). Subsequently, the proportion of variants increased, and the classical P1-2 strain was nearly replaced by variants after 2010. Variant 2a was first reported in 1999 in Japan and became the major P1-2 variant for several years in Asia, but it persisted longer in some European countries (28, 40, 52, 57–59). Variant 2b was first found in 2004 in Germany (36) but did not prevail in Asia (60). Variant 2c was identified in Europe and China around 2010 to 2011 (54, 55, 61) and has become the major P1-2 variant since then in almost all regions (24, 25, 28, 40, 56, 62). In this study, 2c was the major variant (64.6%) from 2012 to 2018, followed by the classical P1-2 (15.0%). This is consistent with the prevalence trends of P1-2 variants in other regions of the world during this period. No P1-1 variants were identified in this study, which is also in agreement with only rare reports of P1-1 variants in the past (53). Thus, it appears that P1-2 may be more prone to homologous recombination in RepMP4 and RepMP2/3 loci, whereas P1-1 keeps a relative stable structure in these two loci. The molecular mechanisms behind this phenomenon merit further investigation. Along with the shift of dominance of P1-2 strains, we expect that more new P1-2 variants will emerge. In this study, two novel P1-2 variants, 2h and 2i, were identified from recent samples (2017 and 2018, respectively). With this obvious change in P1-2 variants, we wonder whether and/or how the change of the P1-2 variants will affect population immunity and eventually shape the pattern of subtype shift.

The MLVA typing scheme for molecular epidemiological analysis of M. pneumoniae has been standardized since its initial development (22, 37, 63). When data from previous studies were reanalyzed using the amended 4-locus typing scheme, three major MLVA types, 4-5-7-2, 3-5-6-2, and 3-6-6-2, were found to have been circulating worldwide from 1962 to 2012 (37, 64). These three major MLVA types were also identified during 2006 to 2013 in the United States: 4-5-7-2 (54%), 3-5-6-2 (32%), and 3-6-6-2 (11%) (16). In this study period (2012 to 2018), the three MLVA types were still dominant but the proportions changed: 4-5-7-2 (35.3%), 3-5-6-2 (41.8%), and 3-6-6-2 (16.6%). Since 2011 to 2014, the proportion of MLVA type 4-5-7-2 has been changing in two directions worldwide, increasing in some European countries (40, 49, 65) and decreasing in Japan and China (24, 30, 62). This change corresponded to the P1 subtype shift in these regions. In this study, MLVA type 4-5-7-2 predominated before 2015; after 2015, MLVA type 3-5-6-2 took its place. This trend also mirrors that of the P1 subtype change in this country (Fig. 1A and 5A). Actually, the correlation between the MLVA types and P1 subtypes has been observed in the United States (16), Asia (24, 26, 30, 46, 66), and Europe (40, 49). We also found a significant association between P1 subtype and MLVA type: the two major MLVA lineages corresponded to the two P1 subtypes; all P1-1 specimens were in MLVA clusters MC1, MC2, and MC4, and all P1-2 specimens were in MLVA cluster MC3 (Table 4, Fig. 4B, and Fig. S3).

A correlation between P1-2 variants and MLVA type was also identified in this study: variants 2a and 2c were mainly MLVA type 3-5-6-2, with a few exceptions; all 2b and 2bv variants were MLVA type 3-6-6-2, and the classical type 2 was mainly MLVA 3-6-6-2 (32/38, 84.2%) (Table 3 and Fig. 4C). This observation is similar to the report from Suzuki et al. (30). There were more MLVA types found in P1-1 than in P1-2 (10 versus 4), suggesting that P1-1 is more genetically heterogeneous than P1-2. On the other hand, within P1-2, the number of variant types is greater than that of MLVA types (7 versus 4), suggesting that P1 variant typing is more discriminating than MLVA typing for subclassification of P1-2 (HGDI of 0.5397 versus 0.4159). The biological meaning/significance of the differing P1 subtype/variants and MLVA types is still unexplored.

We also observed sequence variation in the tandem repeats in locus Mpn13 in one specimen (AccN 72112, TATTATAGTCTATATATTATATATAGTCCTTATTAATAACTATTTTTAT). The identity between the tandem repeats was not high (83%) in this locus in the original report (22). This observed variation suggests that this locus is still under some modifications.

The *p1* AGT trinucleotide repeat variation is significantly associated with P1 subtypes, P1-2 variants, and MLVA types in this study. To our knowledge, this is the first observation of such an association. Some previous studies and our comparative genomics study have identified the *p1* VNTR, but no association with certain genotypes was noticed (18, 55, 67). The association with the P1 subtype might be fundamental to other relationships, as the two P1 lineages are conserved in evolution (18) and MLVA types are correlated with P1 subtypes and P1-2 variant types. In 25 specimens, mixed multiple copy numbers were identified (data not shown), suggesting a common and fast-evolving event happened in this locus. The encoded serine repeat in the P1 protein might be involved in protein conformation changes and glycosylation modifications that affect P1 function (18). It would be interesting to investigate whether *p1* VNTR is correlated with strain pathogenicity.

This study found that MR was associated with the P1-1 subtype; within P1-2 variants, MR was associated with the classical P1-2. However, we did not find a significant association of MR with MLVA types, which was the same as the conclusion in a previous study in the United States (16) but not as those in Asia (30, 46, 68, 69). We noticed that the MR rate within the P1-1 subtype was decreasing from the South/East (24.4%) to the West (5.5%), corresponding to the antimicrobial prescription rates in these regions (14). This observation suggests that development of MR is due to selection of resistant strains under antimicrobial pressure rather than specific genotypes. This agrees with the analysis from Suzuki et al. (30). Given the same antimicrobial pressure, we expect that both subtypes have the same potential to develop resistance. It is noted that in AL, the MR prevalence in P1-2 (18.2%) was higher than that in P1-1 (14.3%). With the type shift from P1-1 to P1-2 in the United States, there will be more P1-2 strains exposed to macrolides. We expect that MR prevalence in P1-2 will increase in the near future.

We also noticed that specimen types affect the distribution of P1-2 variants and *p1* VNTR types but not P1 subtypes and MLVA types. LRT and URT specimen proportions were significantly different among collection sites and years. These factors could be the confounders in the analysis of the M. pneumoniae genotypes. On the other hand, M. pneumoniae identification in LRT or URT might be related to differing strain virulence; thus, a correlation with certain genotypes could truly exist.

Overall, this study revealed that the molecular genotypes of M. pneumoniae between 2012 and 2018 in the United States are diverse and evolving. Certain correlations within M. pneumoniae genotypes were observed. Continued longitudinal prospective monitoring of M. pneumoniae in more locations in the United States is suggested.

## Supplementary Material

Supplemental file 1

## References

[1] Jain S, Williams DJ, Arnold SR, Ampofo K, Bramley AM, Reed C, Stockmann C, Anderson EJ, Grijalva CG, Self WH, Zhu Y, Patel A, Hymas W, Chappell JD, Kaufman RA, Kan JH, Dansie D, Lenny N, Hillyard DR, Haynes LM, Levine M, Lindstrom S, Winchell JM, Katz JM, Erdman D, Schneider E, Hicks LA, Wunderink RG, Edwards KM, Pavia AT, McCullers JA, Finelli L, CDC EPIC Study Team. 2015 Community-acquired pneumonia requiring hospitalization among U.S. children. N Engl J Med 372:835–845. doi:10.1056/NEJMoa1405870. 25714161 PMC4697461

[2] Jain S, Self WH, Wunderink RG, Fakhran S, Balk R, Bramley AM, Reed C, Grijalva CG, Anderson EJ, Courtney DM, Chappell JD, Qi C, Hart EM, Carroll F, Trabue C, Donnelly HK, Williams DJ, Zhu Y, Arnold SR, Ampofo K, Waterer GW, Levine M, Lindstrom S, Winchell JM, Katz JM, Erdman D, Schneider E, Hicks LA, McCullers JA, Pavia AT, Edwards KM, Finelli L, Team CES, CDC EPIC Study Team. 2015 Community-acquired pneumonia requiring hospitalization among U.S. adults. N Engl J Med 373:415–427. doi:10.1056/NEJMoa1500245. 26172429 PMC4728150

[3] Jacobs E, Ehrhardt I, Dumke R 2015 New insights in the outbreak pattern of *Mycoplasma pneumoniae*. Int J Med Microbiol 305:705–708. doi:10.1016/j.ijmm.2015.08.021. 26319941

[4] Lenglet A, Herrador Z, Magiorakos AP, Leitmeyer K, Coulombier D, European Working Group on *Mycoplasma pneumoniae*. 2012 Surveillance status and recent data for *Mycoplasma pneumoniae* infections in the European Union and European Economic Area, January 2012. Euro Surveill 1717:20075. doi:10.2807/ese.17.05.20075-en. 22321134

[5] Eibach D, Casalegno JS, Escuret V, Billaud G, Mekki Y, Frobert E, Bouscambert-Duchamp M, Lina B, Morfin F 2012 Increased detection of *Mycoplasma pneumoniae* infection in children, Lyon, France, 2010 to 2011. Euro Surveill 17:20094. doi:10.2807/ese.17.08.20094-en. 22401503

[6] Oishi T, Takahashi K, Wakabayashi S, Nakamura Y, Ono S, Kono M, Kato A, Saito A, Kondo E, Tanaka Y, Teranishi H, Akaike H, Tanaka T, Miyata I, Ogita S, Ohno N, Nakano T, Ouchi K 2019 Comparing antimicrobial susceptibilities among *Mycoplasma pneumoniae* isolates from pediatric patients in Japan between two recent epidemic periods. Antimicrob Agents Chemother 63:e02517-18. doi:10.1128/AAC.02517-18. 31010867 PMC6591619

[7] Yan C, Sun H, Zhao H 2016 Latest surveillance data on *Mycoplasma pneumoniae* infections in children, suggesting a new epidemic occurring in Beijing. J Clin Microbiol 54:1400–1401. doi:10.1128/JCM.00184-16. 26912752 PMC4844719

[8] Qu J, Yang C, Bao F, Chen S, Gu L, Cao B 2018 Epidemiological characterization of respiratory tract infections caused by *Mycoplasma pneumoniae* during epidemic and post-epidemic periods in North China, from 2011 to 2016. BMC Infect Dis 18:335. doi:10.1186/s12879-018-3250-2. 30016939 PMC6050680

[9] Nir-Paz R, Abutbul A, Moses AE, Block C, Hidalgo-Grass C 2012 Ongoing epidemic of *Mycoplasma pneumoniae* infection in Jerusalem, Israel, 2010 to 2012. Euro Surveill 17:20095. doi:10.2807/ese.17.08.20095-en. 22401504

[10] Kim EK, Youn YS, Rhim JW, Shin MS, Kang JH, Lee KY 2015 Epidemiological comparison of three *Mycoplasma pneumoniae* pneumonia epidemics in a single hospital over 10 years. Korean J Pediatr 58:172–177. doi:10.3345/kjp.2015.58.5.172. 26124847 PMC4481037

[11] Waites KB, Xiao L, Liu Y, Balish MF, Atkinson TP 2017 *Mycoplasma pneumoniae* from the respiratory tract and beyond. Clin Microbiol Rev 30:747–809. doi:10.1128/CMR.00114-16. 28539503 PMC5475226

[12] Okazaki N, Narita M, Yamada S, Izumikawa K, Umetsu M, Kenri T, Sasaki Y, Arakawa Y, Sasaki T 2001 Characteristics of macrolide-resistant *Mycoplasma pneumoniae* strains isolated from patients and induced with erythromycin in vitro. Microbiol Immunol 45:617–620. doi:10.1111/j.1348-0421.2001.tb01293.x. 11592636

[13] Waites KB, Lysynyansky I, Bebear CM 2014 Emerging antimicrobial resistance in mycoplasmas of humans and animals, p 289–322. Caister Academic Press, Norfolk, UK.

[14] Waites KB, Ratliff A, Crabb DM, Xiao L, Qin X, Selvarangan R, Tang YW, Zheng X, Dien Bard J, Hong T, Prichard M, Brooks E, Dallas S, Duffy L, Mixon E, Fowler KB, Atkinson TP 2019 Macrolide-resistant *Mycoplasma pneumoniae* in the United States as determined from a national surveillance program. J Clin Microbiol 57:e00968-19. doi:10.1128/JCM.00968-19. 31484701 PMC6813023

[15] Yamada M, Buller R, Bledsoe S, Storch GA 2011 Rising rates of macrolide-resistant *Mycoplasma pneumoniae* in the central United States. Pediatr Infect Dis J 31:409–410. doi:10.1097/INF.0b013e318247f3e0. 22209916

[16] Diaz MH, Benitez AJ, Winchell JM 2015 Investigations of *Mycoplasma pneumoniae* infections in the United States: trends in molecular typing and macrolide resistance from 2006 to 2013. J Clin Microbiol 53:124–130. doi:10.1128/JCM.02597-14. 25355769 PMC4290910

[17] Zheng X, Lee S, Selvarangan R, Qin X, Tang YW, Stiles J, Hong T, Todd K, Ratliff AE, Crabb DM, Xiao L, Atkinson TP, Waites KB 2015 Macrolide-resistant *Mycoplasma pneumoniae*, United States. Emerg Infect Dis 21:1470–1472. doi:10.3201/eid2108.150273. 26196107 PMC4517703

[18] Xiao L, Ptacek T, Osborne JD, Crabb DM, Simmons WL, Lefkowitz EJ, Waites KB, Atkinson TP, Dybvig K 2015 Comparative genome analysis of *Mycoplasma pneumoniae*. BMC Genomics 16:610. doi:10.1186/s12864-015-1801-0. 26275904 PMC4537597

[19] Diaz MH, Desai HP, Morrison SS, Benitez AJ, Wolff BJ, Caravas J, Read TD, Dean D, Winchell JM 2017 Comprehensive bioinformatics analysis of *Mycoplasma pneumoniae* genomes to investigate underlying population structure and type-specific determinants. PLoS One 12:e0174701. doi:10.1371/journal.pone.0174701. 28410368 PMC5391922

[20] Sasaki T, Kenri T, Okazaki N, Iseki M, Yamashita R, Shintani M, Sasaki Y, Yayoshi M 1996 Epidemiological study of *Mycoplasma pneumoniae* infections in Japan based on PCR-restriction fragment length polymorphism of the P1 cytadhesin gene. J Clin Microbiol 34:447–449. doi:10.1128/JCM.34.2.447-449.1996. 8789036 PMC228818

[21] Spuesens EB, Oduber M, Hoogenboezem T, Sluijter M, Hartwig NG, van Rossum AM, Vink C 2009 Sequence variations in RepMP2/3 and RepMP4 elements reveal intragenomic homologous DNA recombination events in *Mycoplasma pneumoniae*. Microbiology 155:2182–2196. doi:10.1099/mic.0.028506-0. 19389769

[22] Degrange S, Cazanave C, Charron A, Renaudin H, Bebear C, Bebear CM 2009 Development of multiple-locus variable-number tandem-repeat analysis for molecular typing of *Mycoplasma pneumoniae*. J Clin Microbiol 47:914–923. doi:10.1128/JCM.01935-08. 19204097 PMC2668363

[23] Touati A, Blouin Y, Sirand-Pugnet P, Renaudin H, Oishi T, Vergnaud G, Bebear C, Pereyre S 2015 Molecular epidemiology of *Mycoplasma pneumoniae*: genotyping using single nucleotide polymorphisms and SNaPshot technology. J Clin Microbiol 53:3182–3194. doi:10.1128/JCM.01156-15. 26202117 PMC4572556

[24] Xue G, Li M, Wang N, Zhao J, Wang B, Ren Z, Yan C, Wu C, Liu Y, Sun H, Xu M, Sun H 2018 Comparison of the molecular characteristics of *Mycoplasma pneumoniae* from children across different regions of China. PLoS One 13:e0198557. doi:10.1371/journal.pone.0198557. 30138360 PMC6107135

[25] Katsukawa C, Kenri T, Shibayama K, Takahashi K 2019 Genetic characterization of *Mycoplasma pneumoniae* isolated in Osaka between 2011 and 2017: decreased detection rate of macrolide-resistance and increase of p1 gene type 2 lineage strains. PLoS One 14:e0209938. doi:10.1371/journal.pone.0209938. 30682029 PMC6347185

[26] Zhao F, Li J, Liu J, Guan X, Gong J, Liu L, He L, Meng F, Zhang J 2019 Antimicrobial susceptibility and molecular characteristics of *Mycoplasma pneumoniae* isolates across different regions of China. Antimicrob Resist Infect Control 8:143. doi:10.1186/s13756-019-0576-5. 31463046 PMC6708159

[27] Zhao F, Liu J, Shi W, Huang F, Liu L, Zhao S, Zhang J 2019 Antimicrobial susceptibility and genotyping of *Mycoplasma pneumoniae* isolates in Beijing, China, from 2014 to 2016. Antimicrob Resist Infect Control 8:18. doi:10.1186/s13756-019-0469-7. 30697421 PMC6346583

[28] Lee JK, Lee JH, Lee H, Ahn YM, Eun BW, Cho EY, Cho HJ, Yun KW, Lee HJ, Choi EH 2018 Clonal expansion of macrolide-resistant sequence type 3 *Mycoplasma pneumoniae*, South Korea. Emerg Infect Dis 24:1465–1471. doi:10.3201/eid2408.180081. 30014844 PMC6056092

[29] Suzuki S, Konno T, Shibata C, Saito H 2018 Low incidence of macrolide-resistant *Mycoplasma pneumoniae* between April 2016 and March 2017 in Akita Prefecture, Japan. Jpn J Infect Dis 71:477–478. doi:10.7883/yoken.JJID.2018.170. 30175736

[30] Suzuki Y, Seto J, Shimotai Y, Itagaki T, Katsushima Y, Katsushima F, Ikeda T, Mizuta K, Hongo S, Matsuzaki Y 2017 Multiple-locus variable-number tandem-repeat analysis of *Mycoplasma pneumoniae* isolates between 2004 and 2014 in Yamagata, Japan: change in molecular characteristics during an 11-year period. Jpn J Infect Dis 70:642–646. doi:10.7883/yoken.JJID.2017.276. 29093323

[31] Waites KD, Xiao L 2016 Detection of human mycoplasmas and ureaplasmas from clinical specimens by culture and PCR, p 3.15.1.1–3.15.8.5. *In* Leber A (ed), Clinical microbiology procedures handbook. ASM Press, Washington, DC. doi:10.1128/9781555818814.ch3.15.

[32] Waites KB, Xiao L, Duffy LB 2016 Mycoplasma and ureaplasma, p 3 15. *In* Laber A (ed), Clinical microbiology procedure handbook, 4th ed American Society for Microbiology Press, Washington, DC.

[33] Li X, Atkinson TP, Hagood J, Makris C, Duffy LB, Waites KB 2009 Emerging macrolide resistance in *Mycoplasma pneumoniae* in children: detection and characterization of resistant isolates. Pediatr Infect Dis J 28:693–696. doi:10.1097/INF.0b013e31819e3f7a. 19633515

[34] CLSI. 2011 Methods for antimicrobial susceptibility testing of human mycoplasmas. Approved guideline. CLSI document M43-A Clinical and Laboratory Standards Institute, Wayne, PA. 31339681

[35] Cousin-Allery A, Charron A, de Barbeyrac B, Fremy G, Skov Jensen J, Renaudin H, Bebear C 2000 Molecular typing of *Mycoplasma pneumoniae* strains by PCR-based methods and pulsed-field gel electrophoresis. Application to French and Danish isolates. Epidemiol Infect 124:103–111. doi:10.1017/s0950268899003313. 10722137 PMC2810890

[36] Dumke R, Luck PC, Noppen C, Schaefer C, von Baum H, Marre R, Jacobs E 2006 Culture-independent molecular subtyping of *Mycoplasma pneumoniae* in clinical samples. J Clin Microbiol 44:2567–2570. doi:10.1128/JCM.00495-06. 16825381 PMC1489489

[37] Sun H, Xue G, Yan C, Li S, Cao L, Yuan Y, Zhao H, Feng Y, Wang L, Fan Z 2013 Multiple-locus variable-number tandem-repeat analysis of *Mycoplasma pneumoniae* clinical specimens and proposal for amendment of MLVA nomenclature. PLoS One 8:e64607. doi:10.1371/journal.pone.0064607. 23737989 PMC3667773

[38] Dumke R, Jacobs E 2011 Culture-independent multi-locus variable-number tandem-repeat analysis (MLVA) of *Mycoplasma pneumoniae*. J Microbiol Methods 86:393–396. doi:10.1016/j.mimet.2011.06.008. 21704086

[39] Xiao J, Liu Y, Wang M, Jiang C, You X, Zhu C 2014 Detection of *Mycoplasma pneumoniae* P1 subtype variations by denaturing gradient gel electrophoresis. Diagn Microbiol Infect Dis 78:24–28. doi:10.1016/j.diagmicrobio.2013.08.008. 24268834

[40] Gullsby K, Olsen B, Bondeson K 2019 Molecular typing of *Mycoplasma pneumoniae* strains in Sweden, 1996–2017, and the emergence of a new P1 cytadhesin gene, variant 2e. J Clin Microbiol 57:e00049-19. doi:10.1128/JCM.00049-19. 30918047 PMC6535615

[41] Alishlash AS, Atkinson TP, Schlappi C, Leal SM Jr, Waites KB, Xiao L 2019 *Mycoplasma pneumoniae* carriage with de novo macrolide-resistance and breakthrough pneumonia. Pediatrics 144:e20191642. doi:10.1542/peds.2019-1642. 31488697

[42] Hunter PR, Gaston MA 1988 Numerical index of the discriminatory ability of typing systems: an application of Simpson's index of diversity. J Clin Microbiol 26:2465–2466. doi:10.1128/JCM.26.11.2465-2466.1988. 3069867 PMC266921

[43] Harris PA, Taylor R, Thielke R, Payne J, Gonzalez N, Conde JG 2009 Research electronic data capture (REDCap)–a metadata-driven methodology and workflow process for providing translational research informatics support. J Biomed Inform 42:377–381. doi:10.1016/j.jbi.2008.08.010. 18929686 PMC2700030

[44] Kenri T, Okazaki N, Yamazaki T, Narita M, Izumikawa K, Matsuoka M, Suzuki S, Horino A, Sasaki T 2008 Genotyping analysis of *Mycoplasma pneumoniae* clinical strains in Japan between 1995 and 2005: type shift phenomenon of *M. pneumoniae* clinical strains. J Med Microbiol 57:469–475. doi:10.1099/jmm.0.47634-0. 18349367

[45] Zhao F, Liu L, Tao X, He L, Meng F, Zhang J 2015 Culture-independent detection and genotyping of *Mycoplasma pneumoniae* in clinical specimens from Beijing, China. PLoS One 10:e0141702. doi:10.1371/journal.pone.0141702. 26509651 PMC4625007

[46] Sun H, Xue G, Yan C, Li S, Zhao H, Feng Y, Wang L 2017 Changes in molecular characteristics of *Mycoplasma pneumoniae* in clinical specimens from children in Beijing between 2003 and 2015. PLoS One 12:e0170253. doi:10.1371/journal.pone.0170253. 28107399 PMC5249184

[47] Suzuki Y, Seto J, Itagaki T, Aoki T, Abiko C, Matsuzaki Y 2015 [Gene Mutations Associated with Macrolide-resistance and p1 Gene Typing of *Mycoplasma pneumoniae* Isolated in Yamagata, Japan, between 2004 and 2013]. Kansenshogaku Zasshi 89:16–22. doi:10.11150/kansenshogakuzasshi.89.16. 26548292

[48] Ishiguro N, Koseki N, Kaiho M, Kikuta H, Togashi T, Oba K, Morita K, Nagano N, Nakanishi M, Hazama K, Watanabe T, Sasaki S, Horino A, Kenri T, Ariga T, Hokkaido Pediatric Respiratory Infection Study Group. 2016 Regional differences in prevalence of macrolide resistance among pediatric *Mycoplasma pneumoniae* infections in Hokkaido, Japan. Jpn J Infect Dis 69:186–190. doi:10.7883/yoken.JJID.2015.054. 26166502

[49] Kogoj R, Praprotnik M, Mrvic T, Korva M, Kese D 2018 Genetic diversity and macrolide resistance of *Mycoplasma pneumoniae* isolates from two consecutive epidemics in Slovenia. Eur J Clin Microbiol Infect Dis 37:99–107. doi:10.1007/s10096-017-3106-5. 28948376

[50] Pereyre S, Charron A, Renaudin H, Bebear C, Bebear CM 2007 First report of macrolide-resistant strains and description of a novel nucleotide sequence variation in the P1 adhesin gene in *Mycoplasma pneumoniae* clinical strains isolated in France over 12 years. J Clin Microbiol 45:3534–3539. doi:10.1128/JCM.01345-07. 17881549 PMC2168523

[51] Zhang XS, Zhao H, Vynnycky E, Chalker V 2019 Positively interacting strains that co-circulate within a network structured population induce cycling epidemics of *Mycoplasma pneumoniae*. Sci Rep 9:541. doi:10.1038/s41598-018-36325-z. 30679460 PMC6345813

[52] Kenri T, Taniguchi R, Sasaki Y, Okazaki N, Narita M, Izumikawa K, Umetsu M, Sasaki T 1999 Identification of a new variable sequence in the P1 cytadhesin gene of *Mycoplasma pneumoniae*: evidence for the generation of antigenic variation by DNA recombination between repetitive sequences. Infect Immun 67:4557–4562. doi:10.1128/IAI.67.9.4557-4562.1999. 10456900 PMC96778

[53] Dorigo-Zetsma JW, Wilbrink B, Dankert J, Zaat SA 2001 *Mycoplasma pneumoniae* P1 type 1- and type 2-specific sequences within the P1 cytadhesin gene of individual strains. Infect Immun 69:5612–5618. doi:10.1128/iai.69.9.5612-5618.2001. 11500436 PMC98676

[54] Spuesens EB, Hoogenboezem T, Sluijter M, Hartwig NG, van Rossum AM, Vink C 2010 Macrolide resistance determination and molecular typing of *Mycoplasma pneumoniae* by pyrosequencing. J Microbiol Methods 82:214–222. doi:10.1016/j.mimet.2010.06.004. 20547188

[55] Zhao F, Cao B, Li J, Song S, Tao X, Yin Y, He L, Zhang J 2011 Sequence analysis of the p1 adhesin gene of *Mycoplasma pneumoniae* in clinical isolates collected in Beijing in 2008 to 2009. J Clin Microbiol 49:3000–3003. doi:10.1128/JCM.00105-11. 21697320 PMC3147768

[56] Brown RJ, Nguipdop-Djomo P, Zhao H, Stanford E, Spiller OB, Chalker VJ 2016 *Mycoplasma pneumoniae* epidemiology in England and Wales: a national perspective. Front Microbiol 7:157. doi:10.3389/fmicb.2016.00157. 26909073 PMC4754400

[57] Dumke R, Von Baum H, Luck PC, Jacobs E 2010 Subtypes and variants of *Mycoplasma pneumoniae*: local and temporal changes in Germany 2003-2006 and absence of a correlation between the genotype in the respiratory tract and the occurrence of genotype-specific antibodies in the sera of infected patients. Epidemiol Infect 138:1829–1837. doi:10.1017/S0950268810000622. 20334729

[58] Dumke R, Schnee C, Pletz MW, Rupp J, Jacobs E, Sachse K, Rohde G, Capnetz Study Group. 2015 *Mycoplasma pneumoniae* and *Chlamydia* spp. infection in community-acquired pneumonia, Germany, 2011-2012. Emerg Infect Dis 21:426–434. doi:10.3201/eid2103.140927. 25693633 PMC4344269

[59] Edelstein I, Rachina S, Touati A, Kozlov R, Henin N, Bebear C, Pereyre S 2016 *Mycoplasma pneumoniae* monoclonal P1 type 2c outbreak, Russia, 2013. Emerg Infect Dis 22:348–350. doi:10.3201/eid2202.151349. 26812125 PMC4734532

[60] Yamazaki T, Kenri T 2016 Epidemiology of *Mycoplasma pneumoniae* infections in Japan and therapeutic strategies for macrolide-resistant M. pneumoniae. Front Microbiol 7:693. doi:10.3389/fmicb.2016.00693. 27242718 PMC4876131

[61] Spuesens EB, Hartwig NG, van Rossum AM, Vink C 2011 Sequence variation within the P1 gene of *Mycoplasma pneumoniae*. J Clin Microbiol 49:3723. doi:10.1128/JCM.01318-11. 21960704 PMC3187291

[62] Yan C, Xue G, Zhao H, Feng Y, Li S, Cui J, Ni S, Sun H 2019 Molecular and clinical characteristics of severe *Mycoplasma pneumoniae* pneumonia in children. Pediatr Pulmonol 54:1012–1021. doi:10.1002/ppul.24327. 31119869

[63] Chalker VJ, Pereyre S, Dumke R, Winchell J, Khosla P, Sun H, Yan C, Vink C, Bebear C 2015 International *Mycoplasma pneumoniae* typing study: interpretation of *M. pneumoniae* multilocus variable-number tandem-repeat analysis. New Microbes New Infect 7:37–40. doi:10.1016/j.nmni.2015.05.005. 26236493 PMC4501435

[64] Benitez AJ, Diaz MH, Wolff BJ, Pimentel G, Njenga MK, Estevez A, Winchell JM 2012 Multilocus variable-number tandem-repeat analysis of *Mycoplasma pneumoniae* clinical isolates from 1962 to the present: a retrospective study. J Clin Microbiol 50:3620–3626. doi:10.1128/JCM.01755-12. 22952264 PMC3486201

[65] Voronina EN, Gordukova MA, Turina IE, Mishukova OV, Dymova MA, Galeeva EV, Korsunskiy AA, Filipenko ML 2020 Molecular characterization of *Mycoplasma pneumoniae* infections in Moscow from 2015 to 2018. Eur J Clin Microbiol Infect Dis 39:257–263. doi:10.1007/s10096-019-03717-6. 31655931

[66] Whistler T, Sawatwong P, Diaz MH, Benitez AJ, Wolff BJ, Sapchookul P, Thamthitiwat S, Winchell JM 2017 Molecular characterization of *Mycoplasma pneumoniae* infections in two rural populations of Thailand from 2009 to 2012. J Clin Microbiol 55:2222–2233. doi:10.1128/JCM.00350-17. 28490485 PMC5483925

[67] Tian XJ, Dong YQ, Dong XP, Li JY, Li D, Jiang Y, Xin DL 2013 P1 gene of *Mycoplasma pneumoniae* in clinical isolates collected in Beijing in 2010 and relationship between genotyping and macrolide resistance. Chin Med J 126:3944–3948. 24157163

[68] Ho PL, Law PY, Chan BW, Wong CW, To KK, Chiu SS, Cheng VC, Yam WC 2015 Emergence of macrolide-resistant *Mycoplasma pneumoniae* in Hong Kong is linked to increasing macrolide resistance in multilocus variable-number tandem-repeat analysis type 4–5-7–2. J Clin Microbiol 53:3560–3564. doi:10.1128/JCM.01983-15. 26338857 PMC4609705

[69] Lu CY, Yen TY, Chang LY, Liau YJ, Liu HH, Huang LM 2020 Multiple-locus variable-number tandem-repeat analysis (MLVA) of macrolide-susceptible and -resistant *Mycoplasma pneumoniae* in children in Taiwan. J Formos Med Assoc 2020:S0929-6646(19)31151-9. doi:10.1016/j.jfma.2019.12.008. 31924377

