# Supplementary material for: Molecular Characterization of Mycoplasma pneumoniae Isolates in the United States from 2012 to 2018
Source: J Clin Microbiol. 2020 Sep 22;58(10):e00710-20. doi: 10.1128/JCM.00710-20 (PMC7512161; doi:10.1128/JCM.00710-20)
Supplement: Supplemental file 1 [file JCM.00710-20-s0001.pdf]

Supplementary materials:

Supplementary Figure 1. P1 subtype variants. A). Partial sequences of variant 2h and 2i in RepMP2/3 region. Representative sequences of variant 2h (strain 72255) and variant 2i (strain 73192) were aligned with P1-2 reference sequence FH. B). Illustration of the P1 variants.

Supplementary Figure 2. Distribution of *p1* VNTRs with different copy numbers in: A) Upper or lower respiratory (URT or LRT) specimens ($p < 0.001$); B) P1 subtypes ($p = 0.002$); C). P1-2 variants groups ($p = 0.032$); and D). MLVA type groups ($p < 0.001$). * Significant difference after Bonferroni correction.

Supplementary Figure 3. MLVA clustering of the 428 Mp strains by the unweighted pair group method with arithmetic mean (UPGMA) method. The information of the isolates, including isolate name, collection year, state, the clinical sources, P1 subtype, P1-2 variant type, and MLVA type are shown on the right. A cutoff value of 67% similarity was applied to define MLVA clusters (MC, named MC1 to 4). The colors used are based on MLVA clusters.

Supplementary Figure 4. Distribution of macrolide-susceptible and macrolide-resistant *M. pneumoniae* in different states. A). MLVA type 4-5-7-2 ($p = 0.044$). B). MLVA type 3-5-6-2 ($p = 0.001$). C). MLVA type 3-6-6-2 ($p = 0.182$). * Significant difference after Bonferroni correction.

Supplementary Table 1. Summary of the specimens.

Supplementary Table 2. Primers for P1 typing.

Supplementary Table 3. Comparison of the specimen types among different categories.

Supplementary Table 4. Comparison of the distributions of *p1* VNTR among different categories.

Supplementary Figure 1.

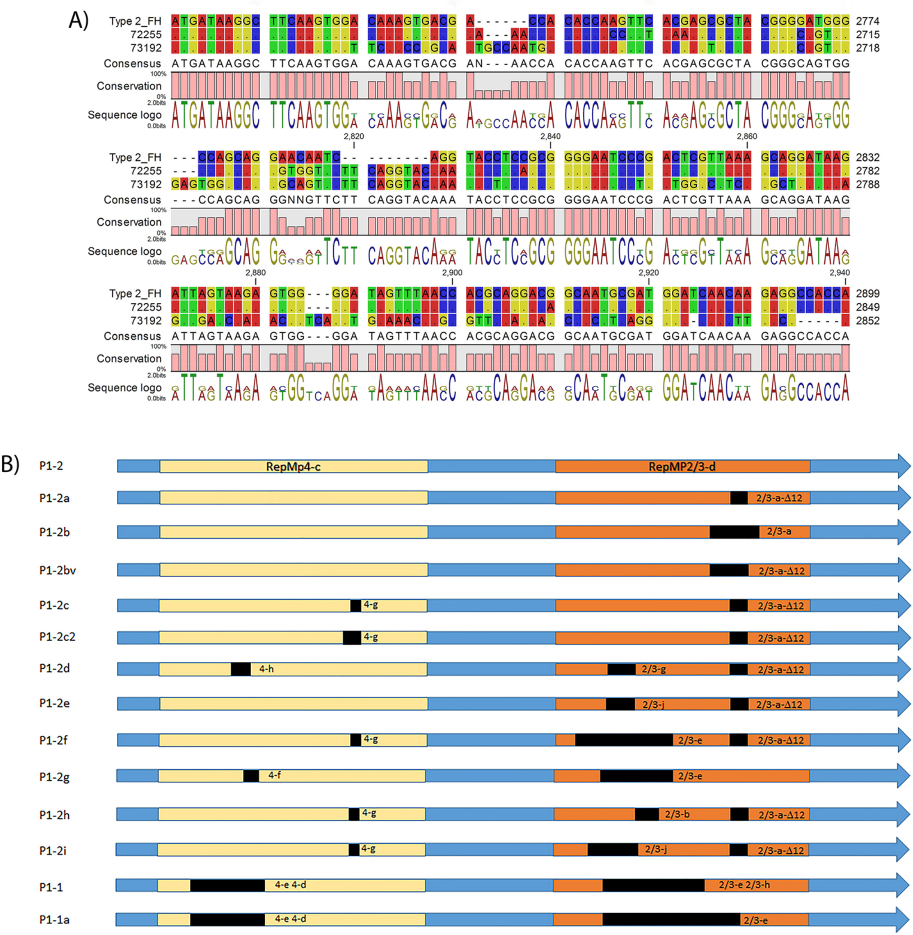

Supplementary Figure 2

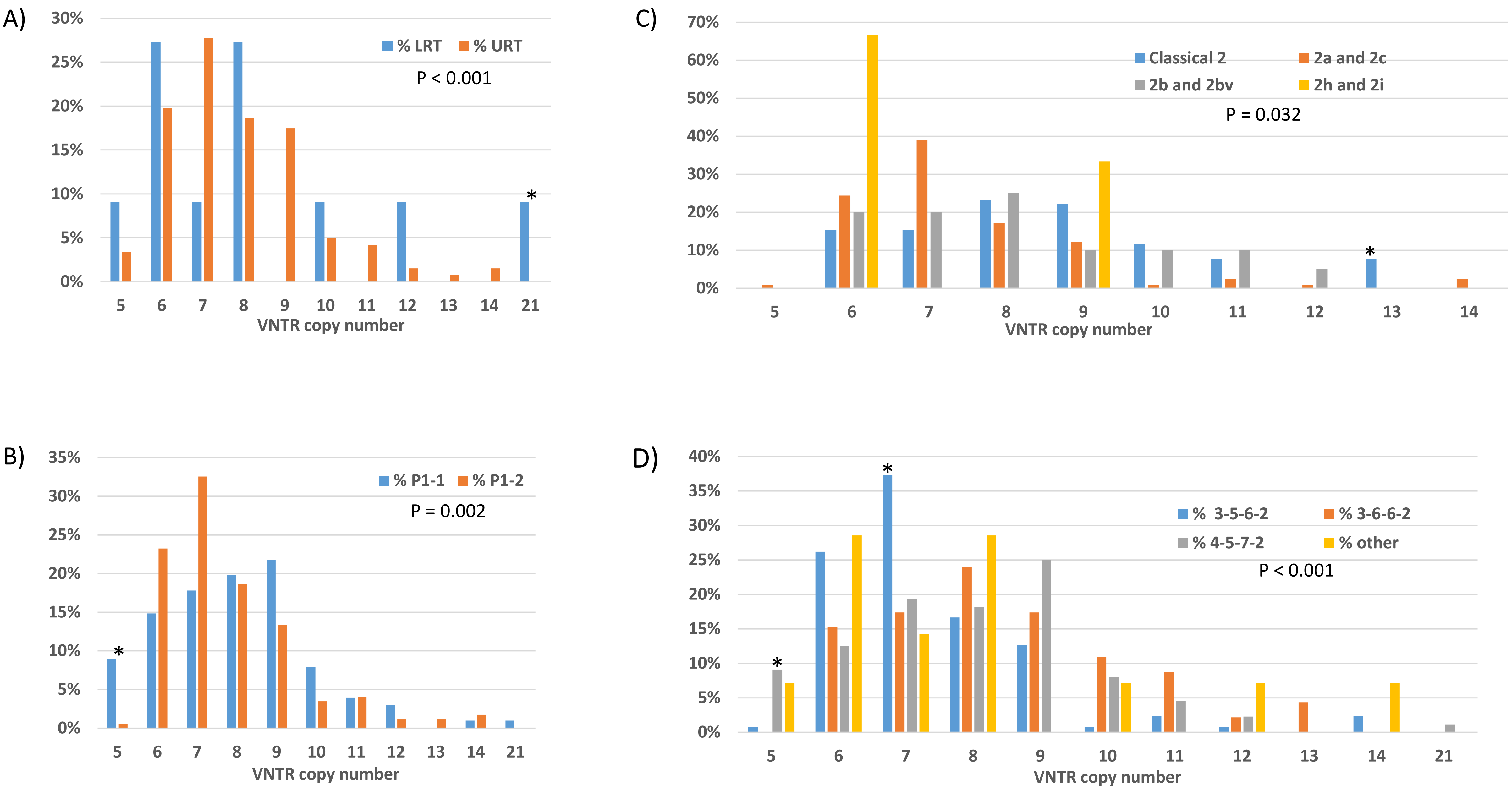

Supplementary Figure 3.

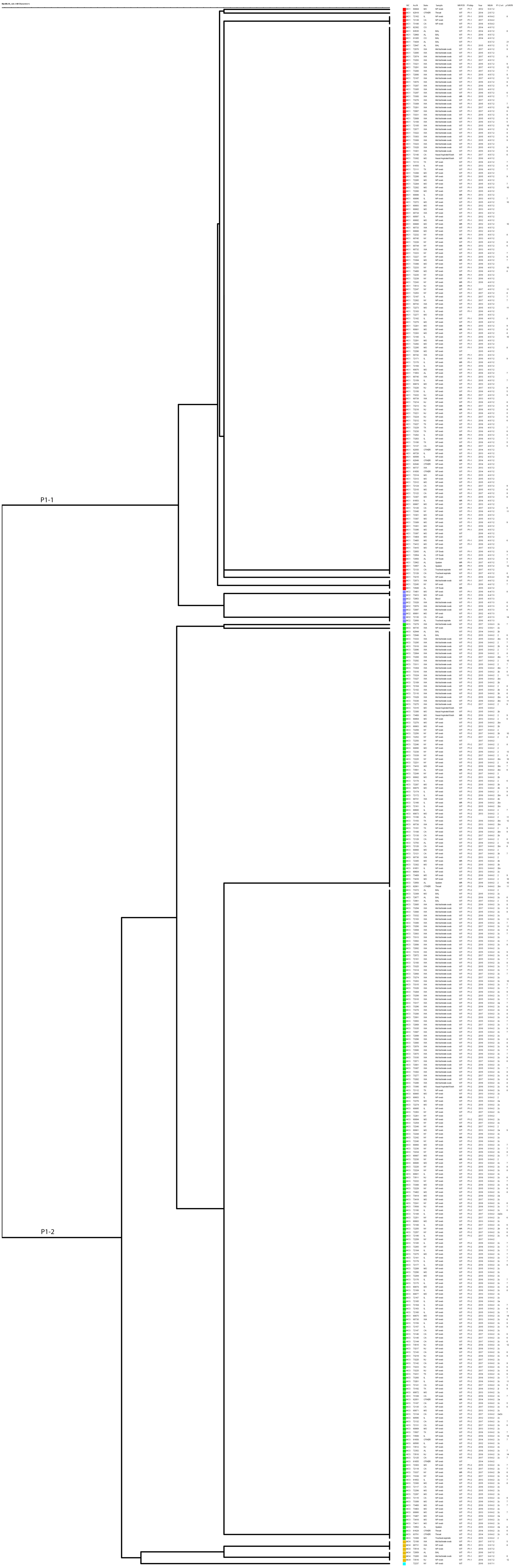

Supplementary Figure 4

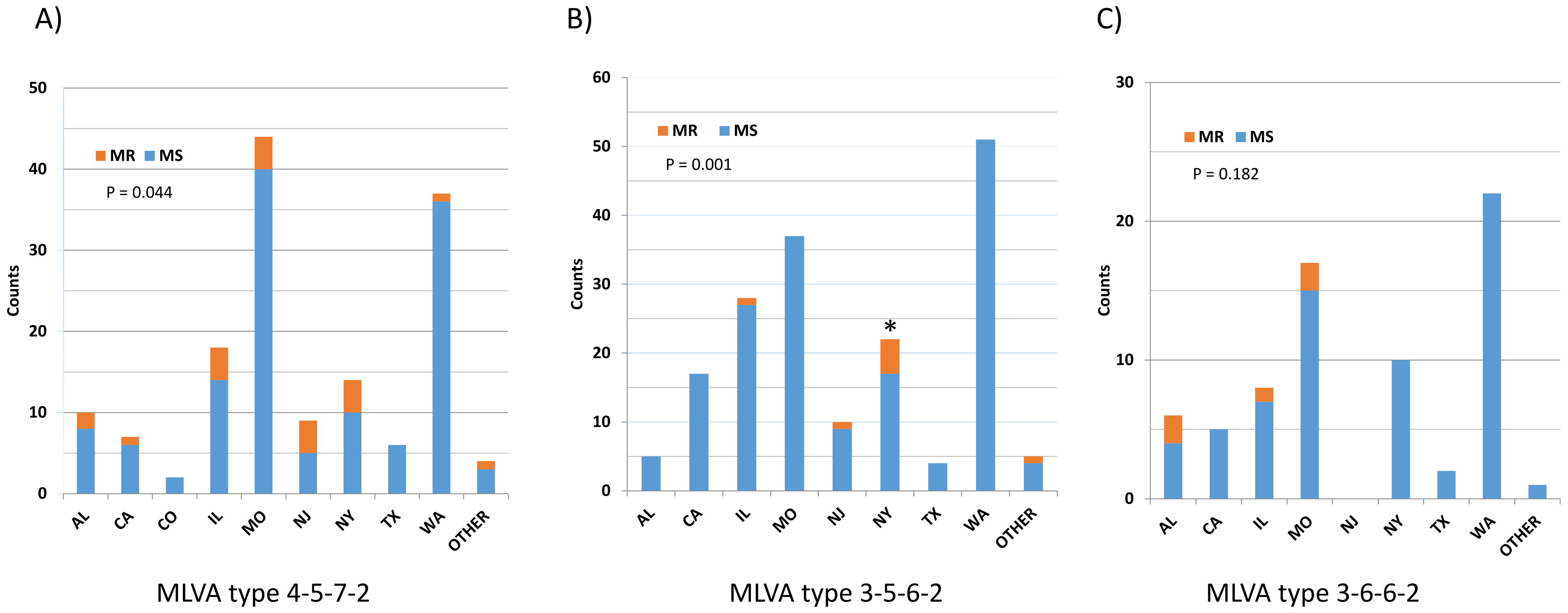

Supplementary Table 1. Summary of the specimens.

Specimens	Frequency	Percentage
Upper respiratory track:	420	94.2%
Mid-turbinate swab	107	24.0%
Nasal Aspirate/Wash	7	1.6%
Nasopharyngeal swab	297	66.6%
Oropharyngeal Swab	4	0.9%
Throat swab	5	1.1%
Lower respiratory track:	24	5.4%
Bronchoalveolar lavage	14	3.1%
Sputum	4	0.9%
Tracheal aspirate	6	1.3%
Blood	1	0.2%
Unspecified	1	0.2%
Total	446	100.0%

Supplementary Table 2. Primers for P1 typing.

Purpose	Primer	Sequence	Position	Amplicon	Reference
First Amplification	ADH1	CTGCCTTGTCCAAGTCCACT	20-39	2031	(1)
	ADH2	AACCTTGTCGGGAAGAGCTG	2031-2050		(1)
	Mp5f	TTGACAAGACCGTCCAATCC	1992-2011	2456	(2)
	Mp16r	TTGGTTGGGTATCTTGATCAGG	4456-4435		(2)
Nested PCR	Mp705	AATGTTTGGCTTGAAGGTGAAG	705-726	949	This study
	ADH3R	GTTGACCATGCCTGAGAACAGTAA	1630-1653		(3)
	Mp11f	CCTCGTTGTCAGTGGCACC	3315-3333	533	(2)
	Mp14r	GCTGGGTGGAATGGTCTGTAC	3847-3827		(2)
Sequencing: ADH1/ADH2	ADH1	CTGCCTTGTCCAAGTCCACT	20-39		(1)
	Mp705	AATGTTTGGCTTGAAGGTGAAG	705-726		This study
	Mp2cf1	TGAAGAACGCCCAAGTGAA	1219-1237		This study
	ADH3R	GTTGACCATGCCTGAGAACAGTAA	1630-1653		(3)
	ADH2	AACCTTGTCGGGAAGAGCTG	2031-2050		(1)
Sequencing: Mp5f/Mp16r	Mp5f	TTGACAAGACCGTCCAATCC	1992-2011		(2)
	Mp6f	GGGAATGGGTACAGGTATGGC	2434-2454		(2)
	Mp7f	AGGCC CCAACTACACCAA	2882-2900		(2)
	Mp19r	GTTTCCGTCACTCGTGCTTG	4038-4019		(2)

Reference:

1. Cousin-Allery A, Charron A, de Barbeyrac B, Fremy G, Skov Jensen J, Renaudin H, Bebear C. 2000. Molecular typing of *Mycoplasma pneumoniae* strains by PCR-based methods and pulsed-field gel electrophoresis. Application to French and Danish isolates. Epidemiol Infect 124:103-11.
2. Dumke R, Luck PC, Noppen C, Schaefer C, von Baum H, Marre R, Jacobs E. 2006. Culture-independent molecular subtyping of *Mycoplasma pneumoniae* in clinical samples. J Clin Microbiol 44:2567-70.
3. Kenri T, Okazaki N, Yamazaki T, Narita M, Izumikawa K, Matsuoka M, Suzuki S, Horino A, Sasaki T. 2008. Genotyping analysis of *Mycoplasma pneumoniae* clinical strains in Japan between 1995 and 2005: type shift phenomenon of M. pneumoniae clinical strains. J Med Microbiol 57:469-75.

Supplementary Table 3. Comparison of specimen types among different categories.

Category	No. of patients	LRT, N (%)	URT, N (%)	P value
All specimens	444	24 (5.4)	420 (94.6)	
States:				<0.001
AL [a]	26	17 (70.8)	9 (2.2)	
CA	36	3 (12.5)	33 (8.1)	
CO [a]	1	1 (4.2)	0 (0)	
IL	58	0 (0)	58 (14.3)	
MO	104	3 (12.5)	101 (24.8)	
NJ	23	0 (0)	23 (5.7)	
NY	48	0 (0)	48 (11.8)	
TX	13	0 (0)	13 (3.2)	
WA [a]	122	0 (0)	122 (30.0)	
Total	431	24 (5.6)	407 (94.4)	
Regions:				<0.001
South/East [a]	97	17 (70.8)	81 (19.9)	
Middle	176	4 (16.7)	172 (42.3)	
West	158	3 (12.5)	155 (38.1)	
Total	431	24 (5.6)	407 (94.4)	
Year:				<0.001
2012	15	0 (0)	15 (3.6)	
2013	52	0 (0)	52 (12.4)	
2014 [a]	18	4 (18.2)	14 (3.3)	
2015	120	6 (27.3)	114 (27.3)	
2016	97	7 (31.8)	90 (21.5)	
2017	92	4 (18.2)	88 (21.1)	
2018	46	1 (4.5)	45 (10.8)	
Total	440	22 (5.0)	418 (95.0)	
Macrolide resistance:				0.439
Resistant	37	3 (12.5)	34 (8.1)	
Susceptible	407	21 (87.5)	386 (91.9)	
Total	444	24 (5.4)	420 (94.6)	
P1 subtype:				0.074
P1-1	169	13 (59.1)	156 (38.9)	
P1-2	254	9 (40.9)	245 (61.1)	
Total	423	22 (5.2)	401 (94.8)	
P1-2 variant (N=254):				0.026
Classical 2 [a]	38	5 (55.6)	33 (13.5)	
2a	8	1 (11.1)	7 (2.9)	
2a/2c	3	0 (0)	3 (1.2)	
2b	18	1 (11.1)	17 (6.9)	

2bv	20	0 (0)	20 (8.2)	
2c	164	2 (22.2)	162 (66.1)	
2h	2	0 (0)	2 (0.8)	
2i	1	0 (0)	1 (0.4)	
Total	254	9 (3.5)	245 (96.5)	
P1-2 variant groups:				0.007
Classical 2 [a]	38	5 (55.6)	33 (13.5)	
2a and 2c	175	3 (33.3)	172 (70.2)	
2b and 2bv	38	1 (11.1)	37 (15.1)	
2h and 2i	3	0 (0)	3 (1.2)	
Total	254	9 (3.5)	245 (96.5)	
MLVA type:				0.792
2-5-7-2	1	0 (0)	1 (0.2)	
3-5-6-1	1	0 (0)	1 (0.2)	
3-5-6-2	179	6 (30)	173 (42.6)	
3-5-6-3	1	0 (0)	1 (0.2)	
3-5-7-1	1	0 (0)	1 (0.2)	
3-5-7-2	3	0 (0)	1 (0.2)	
3-6-6-2	71	3 (15)	68 (16.7)	
3-6-7-2	3	1 (5)	3 (0.7)	
4-4-7-3	2	0 (0)	2 (0.5)	
4-5-3-2	1	0 (0)	1 (0.2)	
4-5-7-2	150	9 (45)	141 (34.7)	
4-5-7-3	6	1 (5)	5 (1.2)	
4-5-8-2	3	0 (0)	3 (0.7)	
4-6-7-2	3	0 (0)	3 (0.7)	
5-5-7-2	1	0 (0)	1 (0.2)	
Total	426	20 (4.7)	406 (95.3)	
MLVA type groups:				0.604
3-5-6-2	179	6 (30)	173 (42.6)	
3-6-6-2	71	3 (15)	68 (16.7)	
4-5-7-2	150	9 (45)	141 (34.7)	
Others	26	2 (10)	24 (5.9)	
Total	426	20 (4.7)	406 (95.3)	

Notes: URT, upper respiratory track; LRT, lower respiratory track; [a]. Significant after Bonferroni correction.

Supplementary Table 4. Comparison of the distribution of *p1* VNTRs among different categories.

Category	Specimen, N (%)	VNTR copy number, N (%)											
		5	6	7	8	9	10	11	12	13	14	21	p value
All specimens	274	10 (3.6)	55 (20.1)	74 (27.0)	52 (19.0)	46 (16.8)	14 (5.1)	11 (4.0)	5 (1.8)	2 (0.7)	4 (1.5)	1 (0.4)	
States:													0.089
AL	18 (6.7)	1	4	4	2	2	1	1	1	1	0	1	
CA	25 (9.3)	3	7	5	3	6	0	0	0	0	1	0	
IL	35 (13.0)	1	5	15	6	5	1	1	0	0	1	0	
MO	39 (14.4)	0	7	11	8	7	4	1	0	0	1	0	
NJ	17 (6.3)	2	4	1	2	4	1	0	2	0	1	0	
NY	31 (11.5)	0	8	6	9	2	3	2	0	1	0	0	
TX	11 (4.1)	1	2	4	1	2	0	0	1	0	0	0	
WA	94 (34.8)	2	16	27	21	18	4	5	1	0	0	0	
Total	270	10 (3.7)	53 (19.6)	73 (27.0)	52 (19.3)	46 (17.0)	14 (5.2)	10 (3.7)	5 (1.9)	2 (0.7)	4 (1.5)	1 (0.4)	
Regions:													0.240
South/East	66 (24.4)	3	16	11	13	8	5	3	3	2	1	1	
Middle	85 (31.5)	2	14	30	15	14	5	2	1	0	2	0	
West	119 (44.1)	5	23	32	24	24	4	5	1	0	1	0	
Total	270	10 (3.7)	53 (19.6)	73 (27.0)	52 (19.3)	46 (17.0)	14 (5.2)	10 (3.7)	5 (1.9)	2 (0.7)	4 (1.5)	1 (0.4)	
Years:													0.079
2012	3 (1.1)	0	0	2	0	0	1	0	0	0	0	0	
2013	15 (5.5)	0	4	5	4	1	0	0	0	0	1	0	
2014	5 (1.8)	0	2	1	1	0	0	1	0	0	0	0	
2015	80 (29.4)	1	12	23	18	19	4	3	0	0	0	0	
2016	60 (22.1)	1	7	20	13	12	4	1	1	1	0	0	
2017	71 (26.1)	4	24	12	12	10	4	3	1	0	1	0	
2018	38 (14.0)	4	6	11	4	4	1	2	3	1	2	0	
Total	272	10 (3.7)	55 (22.2)	74 (27.2)	52 (19.1)	46 (16.9)	14 (5.1)	10 (3.7)	5 (1.8)	2 (0.7)	4 (1.5)	0 (0.0)	
Specimen types:													<0.001
LRT	11 (4.0)	1	3	1	3	0	1	0	1	0	0	1 [a]	
URT	263 (96.0)	9	52	73	49	46	13	11	4	2	4	0 [a]	
Total	274	10	52	73	49	46	13	11	4	2	4	1	
Macrolide resistance:													0.877

Resistant	19 (6.9)	1	4	3	4	4	2	0	1	0	0	0	
Susceptible	255 (93.1)	9	51	71	48	42	12	11	4	2	4	1	
Total	274	10 (3.6)	55 (20.1)	74 (27.0)	52 (19.0)	46 (16.8)	14 (5.1)	11 (4.0)	5 (1.8)	2 (0.7)	4 (1.5)	1 (0.4)	
P1 subtypes:													0.002
P1-1	101 (36.9)	9 [a]	15	18	20	22	8	4	3	0	1	1	
P1-2	173 (63.1)	1 [a]	40	56	32	24	6	7	2	2	3	0	
Total	274	10 (3.6)	55 (20.1)	74 (27.0)	52 (19.0)	46 (16.8)	14 (5.1)	11 (4.0)	5 (1.8)	2 (0.7)	4 (1.5)	1 (0.4)	
P1-2 variants:													0.315
Classical 2	27 (15.6)	0	4	4	6	6	3	2	0	2	0	0	
2a	3 (1.7)	0	0	1	1	1	0	0	0	0	0	0	
2b	7 (4.0)	0	2	1	2	1	1	0	0	0	0	0	
2bv	13 (7.5)	0	2	3	3	1	1	2	1	0	0	0	
2c	120 (69.4)	1	30	47	20	14	1	3	1	0	3	0	
2h	2 (1.2)	0	2	0	0	0	0	0	0	0	0	0	
2i	1 (0.6)	0	0	0	0	1	0	0	0	0	0	0	
Total	173	1 (0.6)	40 (23.1)	56 (32.4)	32 (18.5)	24 (13.9)	6 (3.5)	7 (4.0)	2 (1.2)	2 (1.2)	3 (1.7)	0 (0.0)	
P1-2 variant groups:													0.032
Classical 2	27 (15.6)	0	4	4	6	6	3	2	0	2 [a]	0	0	
2a and 2c	123 (71.1)	1	30	48	21	15	1	3	1	0	3	0	
2b and 2bv	20 (11.6)	0	4	4	5	2	2	2	1	0	0	0	
2h and 2i	3 (1.7)	0	2	0	0	1	0	0	0	0	0	0	
Total	173	1 (0.6)	40 (23.1)	56 (32.4)	32 (18.5)	24 (13.9)	6 (3.5)	7 (4.0)	2 (1.2)	2 (1.2)	3 (1.7)	0 (0.0)	
MLVA types:													<0.001
3-5-6-2	126 (46.0)	1	33	47 [a]	21	16	1	3	1	0	3	0	
3-5-6-3	1 (0.4)	0	0	1	0	0	0	0	0	0	0	0	
3-5-7-2	2 (0.7)	1	0	0	1	0	0	0	0	0	0	0	
3-6-6-2	46 (16.8)	0	7	8	11	8	5	4	1	2	0	0	
3-6-7-2	2 (0.7)	0	1	0	0	0	0	0	1 [a]	0	0	0	
4-4-7-3	1 (0.4)	0	0	0	1	0	0	0	0	0	0	0	
4-5-3-2	1 (0.4)	0	0	0	0	0	1 [a]	0	0	0	0	0	
4-5-7-2	88 (32.1)	8	11	17	16	22	7	4	2	0	0	1	
4-5-7-3	4 (1.5)	0	3	0	0	0	0	0	0	0	1 [a]	0	
4-5-8-2	1 (0.4)	0	0	0	1	0	0	0	0	0	0	0	
4-6-7-2	2 (0.7)	0	0	1	1	0	0	0	0	0	0	0	

Total	274	10 (3.6)	55 (20.1)	74 (27.0)	52 (19.0)	46 (16.8)	14 (5.1)	11 (4.0)	5 (1.8)	2 (0.7)	4 (1.5)	1 (0.4)	
MLVA type groups:													<0.001
3-5-6-2	126 (46.0)	1	33	47 [a]	21	16	1	3	1	0	3	0	
3-6-6-2	46 (16.8)	0	7	8	11	8	5	4	1	2	0	0	
4-5-7-2	88 (32.1)	8 [a]	11	17	16	22	7	4	2	0	0	1	
Others	14 (5.1)	1	4	2	4	0	1	0	1	0	1	0	
Total	274	10 (3.6)	55 (20.1)	74 (27.0)	52 (19.0)	46 (16.8)	14 (5.1)	11 (4.0)	5 (1.8)	2 (0.7)	4 (1.5)	1 (0.4)	

Notes: URT, upper respiratory track; LRT, lower respiratory track; [a]. Significant after Bonferroni correction.
